# The association between disordered eating and psychosis in clinical and non-clinical populations: a systematic review and meta-analysis

**DOI:** 10.1017/S003329172500114X

**Published:** 2025-05-28

**Authors:** Georgia Drymonitou, Amy McCulloch, Sarah Parry, Rhia Gough, Rodrigo Moreira Cruz, Mia Mostoufi, Mariam Jawad, Charlotte Newman, Duncan Harding, Gonzalo Salazar de Pablo, Tom Jewell

**Affiliations:** 1 Oxford Health NHS Foundation Trust, Oxford, UK; 2 Department of Child and Adolescent Psychiatry, Institute of Psychiatry, Psychology and Neuroscience, King’s College London, London, UK; 3 Division of Psychology & Mental Health, University of Manchester, Manchester, UK; 4 Pennine Care NHS Foundation Trust, Manchester, UK; 5 Florence Nightingale Faculty of Nursing, Midwifery & Palliative Care, King’s College London, UK; 6 North East London NHS Foundation Trust, London, UK; 7 South London and Maudsley NHS Foundation Trust, London, UK; 8 Doctoral Programme in Clinical Psychology, University of Hertfordshire, Hatfield, UK; 9 Great Ormond Street Hospital NHS Foundation Trust, London, UK

**Keywords:** eating disorders, disordered eating, psychosis, meta-analysis, prevalence

## Abstract

**Background:**

Eating disorders and psychotic disorders represent two of the most serious psychiatric conditions. Emerging lines of evidence from genetic and epidemiological studies suggest that these disorders may commonly co-occur. This systematic review investigated the association between these disorders across community and clinical populations.

**Method:**

A systematic review was preregistered (CRD42021231771) and conducted according to PRISMA guidelines. Web of Science, PsycINFO and Medline were searched for articles on the association and comorbidity between psychosis and eating disorders up to the 26th February 2024. A random effects meta-analysis was conducted for studies reporting comorbidity of eating disorders and psychotic disorders based on clinical diagnosis or interview measures, to estimate prevalence of the comorbidity between these disorders. A narrative synthesis was conducted for all other studies and grouped by sample (general population, eating disorders or psychotic disorders).

**Results:**

In total 43 studies met inclusion criteria for the systematic review and 16 were included in the meta-analysis. Findings suggest substantial comorbidity between eating disorders and psychotic disorders, with a pooled comorbidity prevalence of 8% (CI: 3, 14) based on clinical diagnosis or interview measures. Studies using self-report questionnaires also highlight the association between eating disorders and psychosis across clinical and community populations.

**Conclusions:**

Eating disorders and psychotic disorders frequently co-occur. Further research should investigate the temporal order of symptom development and consider the need for novel interventions targeted at overlapping psychotic and eating disorder symptoms and associated phenomena.

## Introduction

Eating disorders (EDs) and psychotic disorders constitute two of the most serious psychiatric disorders in terms of their impact on social functioning, comorbidity with physical and mental health conditions, and raised mortality rates (Arcelus, Mitchell, Wales, & Nielsen, 2011; National Institute for Health and Care Excellence, 2014, 2017; Walker, McGee, & Druss, 2015). Both typically emerge between adolescence and emerging adulthood (Solmi et al., 2022) and can be conceptualised as umbrella terms that include a range of diagnoses. Psychotic disorders include but are not limited to schizophrenia, delusional disorder and schizoaffective disorder, whilst EDs include anorexia nervosa (AN), bulimia nervosa and binge eating disorder (World Health Organisation, 2019). In addition, both EDs and psychotic disorders can each be conceptualised as a continuum ranging from subclinical symptoms that are relatively common in the general population (e.g., dieting within certain ranges, hearing voices or seeing visions others do not), to more severe presentations meeting thresholds for diagnostic criteria. Accordingly, incidence and prevalence estimates vary depending on the stringency of criteria. The estimated global incidence of all psychotic disorders is 26·6 per 100 000 person-years (95% CI 22·0–31·7) (Jongsma, Turner, Kirkbride, & Jones, 2019), whilst the estimated lifetime mean prevalence of EDs is 8.4% (Galmiche, Déchelotte, Lambert, & Tavolacci, 2019).

EDs and psychosis have tended to be treated as separate domains in both research and clinical settings. However, several converging lines of evidence suggest that psychosis and EDs are associated in ways that hold promise for future research and treatment. Firstly, emerging evidence from genetic studies suggest a strong genetic association between eating disorders and schizophrenia. Two genome-wide association studies have reported genetic correlations between AN and schizophrenia (Duncan et al., 2017; Watson et al., 2019), meaning that there are genetic variations associated with both disorders. In the study by Watson et al. (2019), genetic correlations were also found between AN, obsessive compulsive disorder (OCD) and bipolar disorder. Meanwhile, Zhang et al. (2021) investigated the familial co-aggregation of EDs and schizophrenia using data from the entire Swedish and Danish population. Individuals with EDs or AN were around 5–6 times more likely to have a diagnosis of schizophrenia compared to individuals without AN, with the odds increasing to 10–13 times more likely in men from the Swedish sample. Relatives of individuals with EDs had increased odds of a schizophrenia diagnosis, with higher odds for relatives with increased genetic relatedness such as siblings and parents.

Recent years have seen increased interest in the notion of a single dimension of psychopathology, or *p* factor, representing a general propensity to developing mental disorders (Caspi, Houts, Fisher, Danese, & Moffitt, 2024). High rates of co-morbidity between psychiatric disorders have led to the suggestion that a more parsimonious structure to psychopathology might exist (Caspi & Moffitt, 2018). One endeavour to investigate the structure of psychopathology is the work of the Hierarchical Taxonomy of Psychopathology (HiTOP) consortium (Conway et al., 2019). In Forbes et al.’s (2021) HiTOP study, a broad thought disorder symptom cluster included eating pathology, psychotic symptoms and OCD, suggesting that eating pathology and psychosis might be more closely associated than has been assumed to be the case. This hypothesis is supported by numerous studies of clinical populations evidencing comorbidity between psychosis and EDs. Indeed, case studies reporting co-morbid psychosis presentations in EDs have been reported for at least 40 years (e.g. Grounds, 1982; Sarró, 2009).

In summary, several converging lines of evidence suggest that the overlap between EDs and psychosis could be fertile ground for clinically relevant developments in research and practice. Genetic studies point to an association between ED and psychosis, raising the possibility of shared aetiological pathways. This could have implications for prevention and early intervention efforts for both disorders. Secondly, mechanisms for the association between ED and psychosis could include nutrition, since nutritional deficiencies are a risk factor for psychosis (Firth et al., 2018). Thirdly, understanding co-morbidity between EDs and psychosis could lead to developments in treatment, such as interventions targeting the ED voice, which is the experience of a critical voice commenting on the hearer’s weight and food intake, instructing them not to eat and telling them that they do not deserve to eat (Dolhanty & Greenberg, 2009; Pugh, 2016). Moreover, antipsychotic medication has a long history of usage in EDs, with recent interest in the potential for olanzapine in adolescent AN (see Lewis, Bergner, Steinberg, Bentley, & Himmerich, 2024, for a review). Finally, it is possible that diagnostic overshadowing may result in under-identification of ED and psychosis within clinical populations, leading to increased complexity and patient distress.

To the best of our knowledge, only two reviews of the ED/psychosis overlap have been conducted. Sankaranarayanan et al. (2021) conducted a systematic review of studies of eating pathology in people with schizophrenia-spectrum disorders, finding 31 studies. This review found elevated rates of ED pathology including disordered eating behaviour (10%–41.5% of patients), binge eating (8.9%–45%) and night eating (4%–30%), with 4.4%–16% of patients meeting criteria for binge eating disorder. More recently, Lo Buglio et al. (2024) conducted a prevalence meta-analysis of eating disorders and disordered eating in individuals at clinical high risk for psychosis, finding a prevalence rate of 5%. Thus far, no review has investigated the association and comorbidity of disordered eating and psychosis in the general population or in the clinical ED population, resulting in a gap in our understanding of how these symptoms co-occur across populations, and how these symptoms have been assessed in research to date.

### Aim

Our aim was to systematically review the evidence for an association between ED and psychosis, across the general population and in clinical ED and psychosis populations.

## Method

Our review was pre-registered in PROSPERO (CRD42021231771). We followed PRISMA guidelines (Page et al., 2021) and our PRISMA checklist for the study is available in Appendix A.

### Searches

Searches were conducted without date restrictions in Web of Science, PsycINFO and MEDLINE up to 26th of February 2024. The search strategy was constructed by GD and TJ and adapted for each database. Free-text and index terms were used to search for literature on eating disorders and psychosis, which were combined using Boolean operators. Search terms for each database are available in Appendix B.

### Screening process

**Figure 1.**
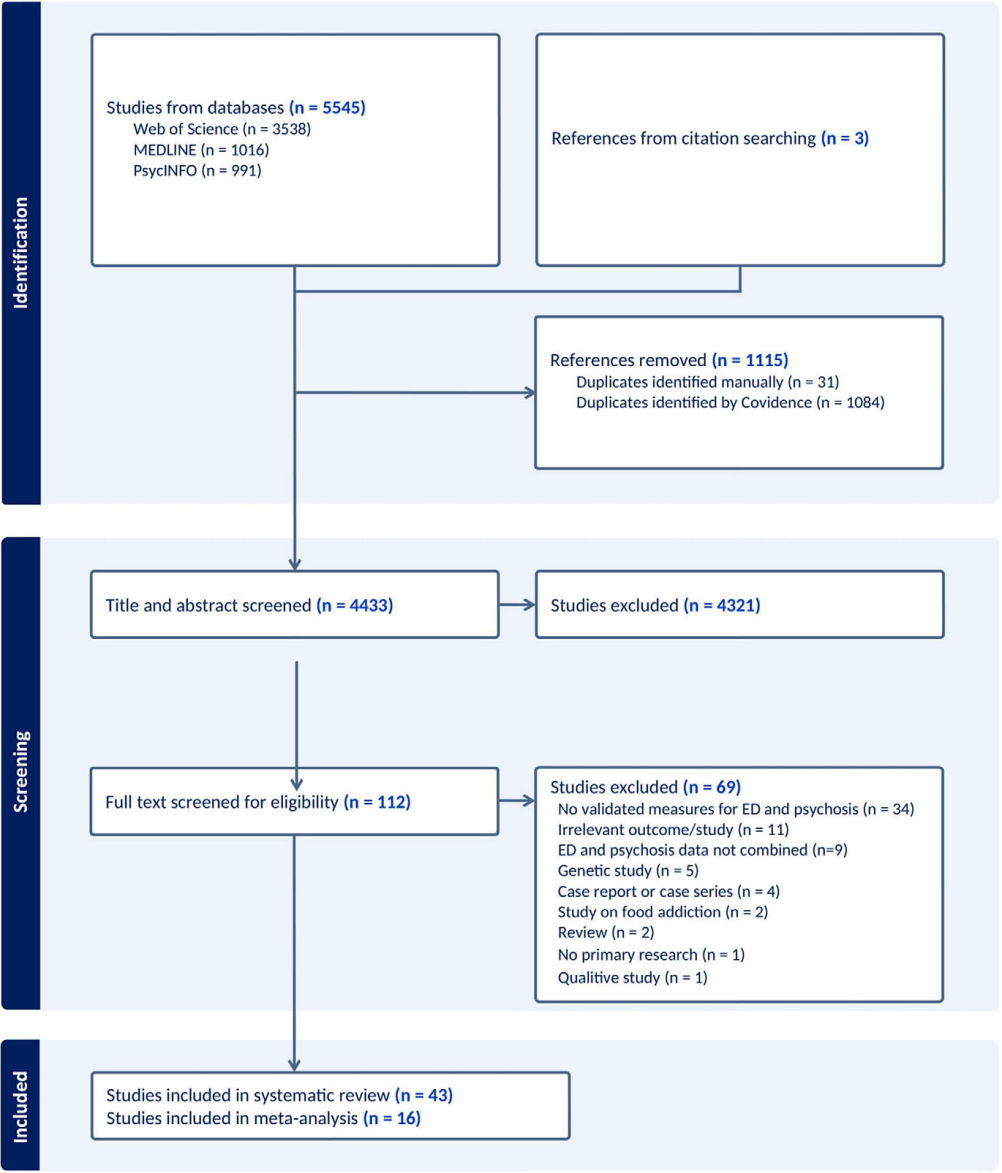
PRISMA flowchart.

Studies were imported into Covidence for screening. RG and RMC independently screened all papers. Any disagreements were resolved by GD and TJ. Our PRISMA flowchart is presented in Figure 1. The reference lists of included studies were manually searched to identify further studies.

### Eligibility criteria

Studies were included in the systematic review if they were: (1) empirical studies with original data, reporting on the comorbidity of ED and psychotic disorder diagnoses, or the association between eating pathology and psychotic symptoms in clinical and non-clinical populations; (2) used validated diagnostic tools, or validated measures of eating pathology and psychotic symptoms, or diagnoses obtained from medical records made using DSM or ICD diagnostic criteria (see Appendix B for details); (3) published in a peer-reviewed journal; (4) in English language. Studies were excluded if they were: (1) qualitative studies; (2) case studies and case series; (3) utilized items derived from scales or interviews without validity data supporting their use; (4) studies reporting genetic correlations.

Studies were included for meta-analysis if they reported co-morbidity of EDs and psychotic disorders using ICD/DSM diagnostic criteria, based on either clinician assessment or use of a validated diagnostic interview.

### Data extraction

Information presented on the methods and results section of the selected studies were extracted independently in pairs by two researchers (GD, AM, SP, MM, MJ and CN), with GD extracting data from all studies. For each study we extracted the following data, as available: the percentage of participants with co-occurring ED and psychotic disorder diagnoses; the percentage of co-occurring symptoms of disordered eating and psychotic symptoms; the association between ED and psychotic disorder symptoms, (e.g. using Pearson’s *r*). The following study and sample characteristics were also extracted: first author, year, country, study design, sample of participants, age, gender, ethnicity/race, socio-economic status, measure of psychosis and eating pathology and summary of main findings. For meta-analysis we extracted: (1) the number of people meeting diagnostic criteria for both EDs and psychotic disorders; (2) the total size of the sample. Where necessary, corresponding authors of eligible studies were contacted for missing data.

### Risk of bias

Risk of bias was assessed using a modified version of the Newcastle-Ottawa Quality Scale (NOS; Wells et al., 2014) for cross-sectional, case-control and cohort studies. Bias was assessed independently in pairs by GD, AM, SP, MM, MJ and CN, with GD assessing all studies. The quality of the studies was assessed on three main domains: the representativeness and selection of the sample, the comparability of the groups/participants and the assessment of outcome/exposure. Studies received a maximum of eight points. Studies were defined as high methodological quality when they received a total score of 7–8 points; as moderate quality when receiving 5–6 points and poor quality when receiving 4 points or below. A modified version of NOS was created in order to assess the risk of bias of cross-sectional studies. Retrospective studies were rated as cross-sectional as their study design matched the NOS criteria for cross-sectional studies. Every study was assessed independently by each reviewer. GSP was consulted for disagreements.

### Data synthesis

We planned to synthesise our findings using narrative synthesis or meta-analysis, as outlined in our preregistration (https://www.crd.york.ac.uk/prospero/display_record.php?RecordID=231771). A decision on performing a meta-analysis was made following completion of data extraction. A meta-analysis of studies reporting co-morbidity of EDs and psychotic disorders using DSM/ICD diagnostic criteria, assessed either using validated measures or by clinical assessment interviews, was possible. A meta-analysis of the correlation between psychosis and EDs was not possible due to lack of consistency in measures and statistical reporting. Therefore, a narrative synthesis was conducted for studies not meeting criteria for meta-analysis, and the findings of the studies were grouped into three categories based on sample, as follows: (1) general population; (2) clinical population with EDs; (3) clinical population with psychotic disorders.

### Meta-analysis

Meta-analysis was performed using the *proportion* function within the *meta* suite of Stata version 18 (StataCorp, 2023) to investigate the pooled prevalence of comorbid psychotic disorders and EDs across clinical and general populations, using a random effects model. The proportion of comorbid cases (referred to as ‘Total successes’ in the forest plot) relative to the total sample (‘Total’) was calculated for each study, along with 95% confidence intervals. To explore sources of heterogeneity, meta-regressions were not indicated due to lack of studies (Deeks, Higgins, Altman, McKenzie, & Veroniki, 2024), but we performed the following subgroup meta-analyses: (1) comorbidity in clinical populations with EDs; (2) comorbidity in clinical populations with psychosis. Meta-analysis of general population studies was not conducted since there were only two eligible studies with unique samples (Convertino et al., 2022; Zhang et al., 2021), with very different methods and populations. The impact of region was explored through meta-analyses of North American and European studies. The impact of study type was explored through meta-analyses of cross-sectional and retrospective studies. No prospective studies were included in the meta-analysis. We investigated heterogeneity using *I*^2^. Publication bias was investigated by visual inspection of funnel plots and Egger’s test. Code and data analysis files are publicly available at https://osf.io/pgk3w/.

## Results

### Study selection

Our database search yielded 5545 studies, of which 1115 were duplicates and were removed. 4433 studies were screened by title and abstract. The full text was accessed for 112 studies, of which 69 were excluded, resulting in 43 studies included in the review, of which 40 studies presented unique data (see Figure 1 for details on study screening and inclusion). Three studies were identified by citation searching. Sixteen studies met the inclusion criteria for meta-analysis. Study and sample characteristics are presented in Tables 1, 2 and 3. References of included studies, and studies excluded at full-text screening stage, are presented in Appendices C and D respectively.

**Table 1.** Study characteristics for general population studies

First author,Year	Country,studydesign	Participants	Agemean, SD	Gender% females	Ethnicity/race	SES	Measure of eating pathology	Measure of psychosis	Main findings (meta-analysis, confidence intervals)
Convertino (2022)	USA, data from the ABCD study, cross-sectional design	11718 (1.6% diagnosed with ED)	9–10 years old	48.8%	Race: 72.6% White, 18.1% Black/African American, 9.3% other Ethnicity: 24.2% Hispanic, 75.8% not Hispanic	Parental education level: 29% graduate, 29.7% college degree, 41.3% other Parental total income: 30% 100,000 or above, 9.1% less than 12,000, 60.9% other	KSADS–5, DSM–5	KSADS–5	The prevalence of current comorbid ED and psychotic disorders was 0.8%, while for past ED and psychotic disorders was 1.5%. The prevalence of current and past hallucination in ED sample was 0.5% and 0.7%. The prevalence of current and past delusions in the ED sample was 2.4% and 5% respectively. The prevalence of any current and past associated psychotic symptoms in ED sample was 3.7% and 3.3% respectively.
Fekih-Romdhane, (2024)	Tunisia, prospective study	510 high school students (at 12 months follow up)	16.1, 1.0	61.2%	NR	2.2% Rural residency: 97.8% Urban residency: Family income: 4.1% <1000 Tunisian dollars (lowest income), 37.8% 3000 (higher), 58.1% other.	EAT–26, MBSRQ-AS	CAPE–42 (only positive symptoms subscale used)	Overweight Preoccupation and Body Area Satisfaction, mediated the prospective association between baseline psychotic experiences and disordered eating 12 months later. Direct effects were significant (*p*<0.001). After accounting for indirect effects through more severe body image concerns at 6 months, higher baseline psychotic experiences were significantly associated with greater disordered eating at 12 months. Baseline psychotic experiences and 6-month body image explained a significant proportion of variance in 12-month disordered eating.
Koyanagi (2016)	UK, cross-sectional study	7307 (5.3% had psychotic-like experience)	NR	NR	87.9% White British 11.5% other	26.9% high, 26.1% medium, 26.5% low, 20.5% other	SCOFF	PSQ	24.7% females and 16.4% males with psychotic like experiences had possible eating disorder. Uncontrolled eating was associated with any psychotic experiences (*p*<0.01) and auditory hallucinations (*p*<0.05) in males. The odds ratio between possible and psychotic like experience was higher in men (*p*<0.001).
Malcolm (2022)	Australia, cross-sectional study	119	31.4, 11.2	100%	Australia/New Zealand (16%), USA/ Canada (29.4%), UK (18.5%), European (25.2%), 10.9% other	NR	Shape Concern and Weight Concern subscales of EDEQ	Paranoia scale	Paranoia was significantly positively associated with both shape (*r*=0.49, *p*<0.001) and weight concerns (*r*=0.42, *p*<0.001).
McGrath (2016)	18 countries from North and South America, Europe, Africa, Asia, Middle East and South Pacific, cross-sectional study	31261 (2385 had lifetime psychotic experience)	NR	NR	NR	NR	CIDI	CIDI	230 people had a lifetime experience of an ED out of 2385 participants with lifetime psychotic experiences (9.6%).
Mohammadi (2020)	Iran, cross-sectional study	27111	NR 6–18 (age range)	51.4%	NR	83.1% urban residence 16.9% rural residence	Persian version of DSM-IV K-SADS-PL	Persian version of DSM-IV K-SADS-PL	No association nor comorbidity was observed between ED and psychotic disorders.
Mutiso (2022)	Kenya, cross-sectional study	9742	21.4, 2.4	46.5%	NR	Marital status: 93.4% Single, 6.6% other 68.6% University level, 31.4% other 21% Lowest wealth index, 16.6% Highest wealth index, 62.3% other	DSM-IV, Subscale of BED from PDSQ	DSM-IV, PDSQ psychosis screen, WERCAP	260 participants met criteria for BED and psychosis (6.4%). The association between BED and psychosis was significant (*r*=0.466, *p*<0.01). The total score on WERCAP psychosis scale was statistically associated with BED (r=.353, *p*<0.01).
Plana-Ripoll (2019)	Denmark, population-based cohort study	5 940 778 (total cohort) (22555 with an ED diagnosis; 86120 with SZ)	32.1, 25.4 (age at start follow-up) 47.0 (age at end follow-up)	50.2%	NR	NR	Psychiatric register, diagnoses based on ICD 8 and 10	Psychiatric register, diagnoses based on ICD 8 and 10	Of the 22555 participants who had a prior eating disorder diagnosis, 1078 individuals (4.78%) developed later comorbid schizophrenia, of whom 997 (92.5%) were women and 81 men (7.5%). Of the 86120 participants who had a prior diagnosis of schizophrenia, 579 (0.67%) individuals developed a later diagnosis of ED.
Rodgers (2022)	United Kingdom, cross-sectional study	7546 (total), 45 had psychosis and 94 had probable psychosis	52.3	59.5%	Psychosis group 84.4% were white, and 15.6% non-White Psychosis probable group: 89.4%Whites and 10.6% non-White	Psychosis group: 11.1% were employed and 88.9% unemployed Psychosis probable group: 10.6% employed and 89.4% unemployed	data from the Adult Psychiatric Morbidity Survey (APMS) 2014	PSQ, SCAN	ED presence in the past year was significantly associated with the co-occurrence of diagnosed psychotic disorder (OR: 12.92, *p*<0.001). ED presence in the past year was statistically significantly associated with probable psychotic disorder (OR: 28.42, *p*<0.001).
Solmi (2018)	United Kingdom, prospective birth cohort study	6361	Data was collected at age 13 and 18	51%	NR	81% mothers were married, 14% single, 5% other. 84% mothers had compulsory education, 16% had degree or higher academic qualification	Youth Risk Behaviour Surveillance System questionnaire (at age 18)	Psychotic-like symptoms interview (at age 13)	29% of children with psychotic experiences at 13 years old had disordered eating at 18 years. Psychotic experiences were associated with greater odds of reporting any disordered eating behaviours (OR: 1.92, *p*<0.0001), and more severe symptoms (OR: 0.58, *p*<0.0001) at age 18 years.
Zhang (2021)	Sweden and Denmark, population-based cohort study	Sweden: 2535191 (12863 AN, 21454 other ED and 3146 SZ), Denmark: 1382367 (7120 AN, 9731 other ED and 6224 SZ)	NR Sweden: 10–36 years age range Denmark: 10–32 age range	NR	NR	NR	Registers and inpatient and outpatient admission records based on ICD and DSM-IV-TR	Registers and inpatient and outpatient admission records based on ICD and DSM-IV-TR	MA: 2% [1, 2].
Zhang (2023)	Sweden, population-based cohort study	12 424 individuals with AN and 20 716 individuals with OED	NR	AN group (94.2%) and in OED (93.7%)	NR	NR	Patient registers, EDE-Q, diagnoses were based on ICD 9,10 and DSM-IV-TR criteria	Patient Register, diagnoses based on ICD criteria	70 (0.56%) individuals with AN and 133 (0.64%) individuals with OED had comorbid diagnoses of schizophrenia during the full follow-up period. ED diagnosis preceding that of schizophrenia in the AN population were 57 (81.4%) and in the OED population (93 (69.9%).

*Note: AN, anorexia nervosa; BED, binge eating disorder; BMI, Body Mass Index; BN, bulimia nervosa; CAPE-42, Community Assessment of Psychic Experiences; CIDI, WHO Composite International Diagnostic Interview; DSM, Diagnostic and Statistical Manual of Mental Disorders; DSM-IV-TR, Diagnostic and Statistical Manual of Mental Disorders, 4^th^ Edition, Text Revision; EAT-26, Eating Attitudes Test; ED, eating disorders; EDEQ, Eating Disorders Examination Questionnaire; ICD, International Classification of Diseases; K-SADS-PL, Schedule for Affective Disorders and Schizophrenia for School-Age Children-Present and Lifetime Version; MA, meta-analysis; MBSRQ-AS, Multidimensional Body Self Relations Questionnaire-Appearance Scales; NR, not reported; OED, other eating disorder; PDSQ, Psychiatric Diagnostic Screening Questionnaire; PSQ, Psychosis Screening Questionnaire; SCAN, Schedules for Clinical Assessment in Neuropsychiatry; SZ, Schizophrenia; WERCAP, Washington Early Recognition Center Affectivity and Psychosis.*

**Table 2.** Study characteristics for eating disorders studies

First author,year	Country,study design	Participants	Agemean, SD	Gender% females	Ethnicity	SES	Measure of eating pathology	Measure of psychosis	Main findings(Meta-analysis, confidence intervals)
Blinder, 2006	USA, cross-sectional study	2436 (520 AN-R; 436 AN-BP; 882 BN; 598 EDNOS)	23.4, 8.6	100%	95.2% White, 2.9% Hispanic, 0.2% Black, 1.7% other	Education level: 30% < high school, 15% college, 55% other Marital status: 18% married, 72% unmarried, 10% divorced	DSM-IV, EDI–2	DSM-IV, SCID-I	MA: 0% [0, 1]
Camprodon-Boadas, 2023	Spain, case-control study	60 (20 AN, 20 OCD, 20 FEP).	14.9, 1.6 AN, 14.1, 1.9 OCD, 15.7, 1.3 FEP	100% AN, 55% OCD, 60% FEP	NR	NR	Diagnosis based on DSM–5, K-SADS-PL (all groups) EAT–40 and drive for thinness subscale of EDI–2 (AN group)	BABS (all groups) PANSS (FEP group)	Patients with OCD scored significantly lower in all scale items apart from the fixation of ideas compared to AN and FEP. AN and FEP group scored higher on the delusionality and conviction of their belief compared to the OCD group (*p*<0.001). There were no significant differences between the FEP and AN group apart from the delusions of reference that FEP group scored higher.
Catone, 2021	Italy, cross-sectional study	92 (66AN; 26 OSFED)	14.3, 1.9	85.9%	NR	NR	Diagnoses based on DSM–5, EAT–26	Paranoia subscale of SPEQ	Twenty patients (21.7%) had score on paranoia scale ≥ 38. There was high and significant correlation between paranoia and disordered eating symptoms (*r*=0.623, *p*<0.001). Regression analysis demonstrated that social anxiety, depression and body image are confounders of the correlation.
De Young, 2022	USA, prospective observational study	50 AN (27 AN-R 23 AN-BP)	29.9, 10.5	100%	94% White, 2% Asian, 4% other	NR	DSM–5, MAEDS, CIA, BCQ	BABS interview	One patient (2%) had a comorbid psychotic disorder. Nine (18.0%) patients met criteria for beliefs of delusional intensity, and 17 (34.0%) met criteria for poor insight. Patients with delusional beliefs and poor insight experienced more fear of fatness and restrictive eating (*p*<0.001). There was no significant change from admission to discharge on the proportion of people with delusional beliefs (20%) and poor insight (29%).
Grilo, 1996	USA, case-control study	136 (31 ED inpatients – 11 AN; 9 BN; 11 EDNOS)	19.8, 6.5 (ED), 20, 4.4 (patients without ED)	100%	ED patients: 90% Caucasians, 6% African Americans, 4% other Comparison group: 86% Caucasians, 10% African American, 4% other	Socioeconomic status: 3, 1.3 (ED) lower to upper middle class 3.4, 1.4 (comparison) lower to upper middle class, Marital status: 100% single (ED), 90% single, 10% other (comparison) Employment status: 23% unemployed, 77% other (ED), 15% unemployed, 85% other (comparison)	KSAD, SCID, DSM-III-R	KSAD, SCID, DSM-III-R	MA: 13% [3, 27].
Herzog, 1992	USA, cross-sectional study	229 (41 AN; 98 BN; 90 mixed AN+BN)	22.8, 7.4 AN; 24.8, 6.1 BN; 26.1, 6.6 AN+BN	100%	97% Caucasian, 1.3% Black, 0.8% Asian, 0.9% other	Socioeconomic status: 52% comfortable, 19.7% just enough for living, 28.3% other Marital status: 83% single, 9.6% married, 7.4% other	DSM-III-R criteria, EDI, EAT-SADS-L	SCL–90, SADS-L	MA: 2% [0, 4].
Hudson, 1984	USA, cross-sectional study	130 (20 AN; 40 AN+BN; 70 BN)	27.6, 8.4	95%	NR	NR	DSM-III criteria	DIS, DSM-III	MA: 2% [0, 5].
Kambanis, 2023	USA, cross-sectional study	50 AN (27 AN, restricting type and 23 AN, binge eating/purging type)	30.4, 10.8	100%	96% White/ Caucasian, 4% other	NR	DSM–5, MAEDS, CIA, BCQ	BABS interview	18% met criteria for beliefs of delusional intensity and 34% for beliefs of poor insight. Most of the beliefs were body image (64%) and food (30%) related. Higher scores on BABS were associated with more body-checking behaviours (*r*=0.377, *p*<0.01), purgative behaviours (*r*=0.340, *p*<0.01), restricting eating (*r*=0.519, *p*<0.01) and fear of fatness (*r*=0.473, *p*<0.01).
Konstantakopoulos, 2012	Greece, cross-sectional study	72 (39 AN (22 restricting type and 17 binge-purge subtype); 33 BN)	AN-R 24.1, 5.6; AN-BP 25.3, 4.9; BN 26.5, 6.1	100%	NR	Education: (mean years, SD) AN-R 14.7, 1.6, AN-BP 14.8, 1.4, BN 14.8, 1.7	DSM-IV, EAT–26, BITE, EDI	BABS, PANSS, GIR	Eleven patients with AN (28.2%) were classified as having delusional body image beliefs. Delusional beliefs were significantly more frequent in the AN-R group (45.5%) than both the AN-BP (5.9%) (χ2=7.42, *p*=0.006) and the BN (χ2=18.33, *p*<0.001) groups. There was no significant difference between AN-BP and BN groups (χ2=2.13, *p*=0.345).
Konstantakopoulos, 2020	Greece, case-control study	88 (46 AN (29 AN-R; 17 AN-BP); 42 healthy controls)	NR	100%	NR	Education (median year): AN 15.5, Controls 15	SCID clinical interview DSM-IV-TR, EAT–26	BABS	Twelve out of 46 patients with AN (26.1%) were classified as having delusional body image beliefs and 30.4% (14/46) as having overvalued ideas. Six controls (14.3%) were categorized as having overvalued ideas while none had a delusional belief.
Lysaker, 2023	Russia, case-control study	133 (80 ED – 40 AN and 40 BN; 53 SSD – 40 SZ and 13 schizoaffective)	34.2, 8.9 SSD, 23.95, 4.9 AN, 24.1, 5.6 BN	56.6% SSD, 100% AN, 100% BN	NR	NR	Diagnostic clinical interview based on ICD–10, EAT–26	Diagnostic clinical interview based on ICD–10, PANSS	The mean score of the SSD group on the EAT–26 scale was 7.23 (8.07), which is below the clinical threshold. The mean score on the total PANSS in SSD group was 70.64 (10.93) which was higher compared to the AN group 62.78 (SD 8.34) and BN group 47.90 (5.84). AN group had higher scores in all the subscales and total PANSS scares compared to BN group.
Mensi, 2020	Italy, cross-sectional study	94 (76 AN and 18 not otherwise specified)	15.1, 2.3 (HR-); 15.5, 1.3 (HR+)	100%	NR	The SES index is according to Hollingshead range (0–90), mean and SD provided: 38.0, 16.1 HR−; 31.7, 14.2 HR+ Percentage of separated Parental marital status: 33.3% HR−, 15.2% HR+	DSM–5, MROAS, EDI–3	CAARMS	MA: 4% [1, 9].
Miotto, 2010	Italy, case-control study	743 (112 ED –61 AN (47 AN-R and 14 AN-BP); 51 BN (12/51 non-purging); 631 control group)	22.3, 7.4 AN; 24.0, 6.7 BN; 16.5, 1 control group	100%	NR	Mean and SD scores for SES provided which reflect on rooms available per person at home: 2.2, 1.9 AN; 2.3, 1.1 BN; 2.0, 0.8 controls	DSM-IV, EAT–40, BITE, BAT	SCL–90R	No case of comorbid schizophrenia was found among the ED patients. 11 patients with AN (18.0%) and 9 with BN (17.6%) endorsed just one symptom/item of psychosis, while 47.6% AN patients and 66.7% BN patients endorsed 2 or more psychosis symptoms.
Mountjoy, 2014	Australia, case-control study	70 (27 SZ, 20 ED, 23 controls)	38.3, 8.8 (SZ), 25.6, 7.4 (AN), 24.6, 7.1 (control)	25.9% (SZ), 100% (AN), 100% (control)	NR	Mean years of education: 11.8 ±2.7 (SZ), 14 ±2.7 (AN), 14.7±1.8 (controls)	DSM-IV-TR, MINI	DSM-IV-TR, MINI, BABS, PDI, PSYRATS	The rate of unusual beliefs was higher in AN and SZ groups. The prevalence of delusional beliefs was 10% in the AN group, 37% in SZ and 4% in control group. The percentage of poor insight about the dominant belief was 30% in AN, 26% in SZ and 4% in control group. The preoccupation and distress in relation to the dominant belief was higher in the AN group compared to SZ and control groups (*p*<0.001).
Patel, 2018	USA, cross-sectional study	3319 inpatients with BN	NR	92.5%	81.4% White, 6.7% Hispanic, 5% Black, 6.9% other	NR	ICD–9-CM diagnoses	ICD–9-CM diagnoses	MA: 52% [51, 54].
Pruccoli, 2023	Italy, prospective observational study	163 patients with FED (133 AN, 17 BN, 9 BED, 4 UFED)	17.2, 3 (FED)	93.3% (FED)	NR	NR	DSM–5, EDRC, EDI–3, BUT-GSI	SCL–90-R	Patients with AN had a mean score of 53.4 (±11.1) on paranoia scale at admission and 50.7 (±12.2) at discharge. They had a mean score of 58.0 (±11.3) on psychoticism scale at admission and 52.9 (±12.2) at discharge. There was significant reduction in paranoia (*p*= 0.005) and psychoticism (*p*<0.001) from admission to discharge. Patients with higher EDI–3 EDRC baseline scores presented, at discharge, higher score on paranoia, and psychoticism.
Steinglass, 2007	USA, cross-sectional study	25 patients with AN (7 without amenorrhea)	24.6, 8	100%	NR	NR	SCID-I, DSM-IV, EDE, EDI	BABS; PANSS (was administered to 11 participants)	20% met criteria for delusional beliefs and 28% for poor insight. The most common dominant beliefs were related to shape/weight fear (17.7%) and fear of losing control (4.2%). No correlation between BABS total score and delusional participants with their clinical characteristics (BMI, duration of illness, age) was found. BABS total score was significantly correlated with the drive for thinness scale of (*r*=0.41, *p*=0.04). Significant differences in PANSS score among delusional (n=2) and non-delusional (n=9) participants were reported, with participants classified as delusional having higher scores (*p*= 0.033).
Striegel-Moore, 1999	USA, case-control cross-sectional study	322 (161 ED veterans, 161 controls) (161 ED: 63 females: 11 AN, 20 BN, 7 AN and BN, 25 EDNOS; 98 males: 25 AN, 17 BN, 56 EDNOS)	Female: ED 35.3, 9.8; control 51.6, 17.1 Males: ED 53.6, 15.0 Control 60.3, 14.1	39.1%	Females: 84.1% Caucasian, 15.9% other Males: 80.6% Caucasian, 19.4% other	NR	ICD–9-CM codes	ICD–9-CM codes	MA: 20% [15, 27].
Valente, 2017	Italy, cross-sectional study	267 ED patients	37.3, 12.5 (total population)	83.5%	NR	Education level: 52.3% high school diploma, 47.7% other Marital status: 51.3% single, 48.7% other Occupation: 57.6% occupied, 42.4% other	DSM-IV-TR SCID-II, BUT	SCID-II	MA: 3% [1, 5].

*Abbreviations: BAT, Body Attitudes Test; BCQ, Body Checking Questionnaire; BITE, Bulimic Investigatory Test of Edinburgh; BUT-GSI, Body Uneasiness Test Global Severity Index; CAARMS, Comprehensive Assessment of At-Risk Mental States; CIA, Clinical Impairment Assessment, CIDI, WHO Composite International Diagnostic Interview; DIS, National Institute of Mental Health Diagnostic Interview schedule; DSM-5, Diagnostic and Statistical Manual of* Mental Disorders 5th Edition; DSM-IV-TR, Diagnostic and Statistical *Manual* of Mental Disorders, 4th Edition, *Text Revision; EAT, Eating Attitudes Test; EDE, Eating Disorder Examination; EDI-3, Eating Disorder Inventory; EDNOS, Eating Disorders Not Otherwise Specified; EDRC, Eating Disorder Risk; FED, Feeding and Eating Disorders; FEP, First-Episode of Psychosis; HR−, no risk for psychosis; HR+, at high risk for psychosis/presence of subthreshold psychosis; ICD-9-CM, International Classification of Disease, Ninth Revision Clinical Modification; KSAD, Schedule for Affective Disorders and Schizophrenia for School-Aged Children – Epidemiologic Version; K-SADS-PL, Children-Present and Lifetime Version; MA, meta-analysis; MAEDS, Multifactorial Assessment of Eating Disorder Symptoms; MROAS, Morgan-Russell Outcome Assessment Schedule; NR, not reported; OCD, obsessive-compulsive disorder; OSFED, other specified feeding or eating disorder; PSQ, The Psychosis Screening Questionnaire; SADS-L, Schedule for Affective Disorders and Schizophrenia; SADS-L, modified version of Schedule for Affective Disorders and Schizophrenia Lifetime version to include a section for DSM-III-R eating disorders; SCAN, version 2.1, Schedules for Clinical Assessment in Neuropsychiatry; SCID, Structured Interview for DSM-III-R; SCID-I, Structured Clinical Interview for DSM-IV Screen Patient Questionnaire-extended; SCID-I, Structured Clinical Interview for DSM-IV Axis I disorders; SCID-II, Structured Clinical Interview for DSM-IV Axis II; SCL-90-R, revised Hopkins Symptom Checklist; SCL-90-R, symptom Check List-90-R; SPEQ, Specific Psychotic Experiences Questionnaire; UFED, unspecified feeding or eating disorders.*

**Table 3.** Study characteristics for psychotic disorders studies

Firstauthor,year	Country,study design	Participants	AgeMean, SD	Gender% females	Ethnicity	SES	Measure of eating pathology	Measure of psychosis	Main findings(Meta-analysis, confidence intervals)
Civil Arslan, 2015	Turkey, cross-sectional study	158 (68 SZ, 12 schizoaf-fective, 78 bipolar disorder)	39.7, 10.3	43.7%	NR	Mean years of education: 9.4 ± 3.7 Marital status: 41.1% married, 48.7% single, 10.2% other	NEQ, Clinical interview based on NES criteria	SCID-I, PANSS	MA: 5% [1, 11].
Fawzi, 2012	Eygpt, case-control study	100 (50 SZ, 50 controls)	29.4, 10.2 SZ, 31.1,10,8 controls	42% SZ and control	NR	Marital status SZ: 58% unmarried, 42% married Controls: 40% unmarried, 60% married Employment SZ: 64% unemployed, 36% employed; Controls: 44% unemployed, 56% employed	EAT–40	Clinical interview, DSM-IV, PANSS	Patients with schizophrenia had an EAT40 mean score (23.4 ± 7.8) higher than that of controls (19.7 ± 7.2) (*p*=0.015). Disordered eating was two and a half times more prevalent (*p*=0.027) in SZ (30%) compared to controls (12%). Disordered eating was associated higher scores on PANSS total score (Z= −2.046; *p*= .041), and positive symptoms (z= −3.168; *p* = .002).
Khazaal, 2006	Switzerland, case-control study	80 (40 SZ patients, 40 controls)	31.7 ± 9.2 SZ with BMI<28; 36.1 ± 8.9 SZ with BMI ≥ 28. 21.1 ± 2.5 controls with BMI<28; 33.8 ± 4.9 controls with BMI ≥ 28.	47.5% SZ, 52.5% controls	NR	NR	SCID-IV, DSM-IV	DSM-IV	MA: 23% [11, 37].
Khosravi (2020)	Iran, case control study	308 (154 SZ – 83 were in active phase; 71 in remission, 154 controls)	NR	56.6% active SZ, 60.6% remission SZ, 45.5% control	NR	Marital status Active phase SZ: 50.6% married, 49.4% single, remission SZ: 49.3% marries, 50.7% single, Controls: 57.1% married, 42.9% single Education level Active SZ: 26.5% high school, 73.5% other, Remission SZ: 23.9% high school, 76.1% other, Controls: 22.1% high school, 77.9% other.	EAT–26	SCID–5-RV, DSM–5 diagnoses, PANSS	The prevalence of disordered eating behaviours (defined by a total score of ≥20 on EAT–26) was 41.5% in SZ and 10.3% in controls (*p* = 0.012).
Lyketsos, 1985	Greece, case-control study	219 (137 SZ, 22 inpatients with affective disorders and 60 controls)	49.6 median age (SZ) mean NR	58.6% (SZ), 77.2% (affective), 60% (control)	NR	Social class SZ: 83.2% lower class, 16,8% other, affective group: 72.7% lower class, 27.3% other, controls: 90% lower class, 10% other	EAT, DSM-III, Schizophre-nic eating disorders questionnaire	DSM-III, Schizo-phrenic eating disorders questionnaire	MA: 1% [1, 3].
Malaspina, 2019	USA, cross-sectional study	288 SZ	32.7	36.8%	NR	NR	DIGS, eating disorder assessment, DSM-IV-TR	DIGS, PANSS, DSM-IV-TR	MA: 9% [6, 13].
Palmese, 2013	USA, cross-sectional study	100 SZ	46.5, 10	61%	43% Caucasian 49% African American, 5% Hispanic, 3% other	NR Mean years of education: 12.7 ±3 SD	NEQ, Semi-structured interview based on NES criteria	SCID, PANSS, DSM-IV-TR	MA: 12% [6, 19].
Salazar de Pablo, 2020	USA, cross-sectional study	248 (65 met APS criteria)	15.4, 1.5	69.4%	54.6% White, 18.1% Black/African American, 27.3% other	NR	SCID, K-SADS-PL, DSM–5	SCID, K-SADS-PL, SIPS/SOPS	MA: 15% [7, 25].
Salvatore, 2021	USA, prospective observational study	329 (216 patients with bipolar-I, 71 schizoaffective, 42 psychotic major-depressive	31.5 bipolar-I, 39.3 major depression, 26.1 schizoaffective	46.3 % bipolar-I, 57.1% major depression, 26.8 % schizoaffective	NR	NR	SCID-P based on DSM-III-R, DSM–5,	SCID-P based on DSM-II-R, DSM–5, SAPS, SANS, BSABS, AMDP system	Prodromal features of eating disorders were present in 15.5% of participants later diagnosed with schizoaffective disorder.
Saraçli, 2015	Turkey, cross-sectional study	433 (47 patients with psychotic disorders)	37.8, 12.0	70.7%	NR	65.8% married, 34.2% other	NEQ, clinical self-report based on NES criteria	DSM-IV, SCL–90R	MA: 7% [3, 13].
Stein, 2005	Israel, cross-sectional study	30 SZ	70, 6.5	100%	Around 2/3 SZ were born in Eastern Europe, 20% in North Africa and the rest were from Israel	NR Mean years of education was 9.1±5.5	SCID-I/P, EAT–26	SCID-I/P, DSM-IV, PANSS	Four patients (13.3%) scored above 20 on EAT26 (35.8±13.1). These patients had higher score on positive (14.8±10.0 vs. 7.7±9.3) PANSS subscale and the total PANSS score (83.8+14.4) compared to the participants with EAT<20 scores (71.1+16.7).
Teh, 2021	Singapore, cross-sectional study	329 (156 patients with SZ, 173 depression and substance use disorders)	29.6 ± 5.6 (SZ), 27.6 ± 5.8 (depression and substance disorders patients)	48.3%	68.4% Chinese 17% Malay, 10.3% Indian, 4.3% other	Marital status: 83.3% unmarried, 16.7% other Educational level: 1.8% primary/no education, 30.7% secondary, 67.5% other	EAT–26	DSM-IV, Medical records	Patients with SZ had lower total mean scores on EAT–26 (8.8±8.2) compared to patients with depression and substance use disorders (10.9±9.0). 11.3% SZ patients scored above threshold in EAT26 (scores of >= 20) and self-reported high risk of disordered eating. SZ with disordered eating had greater scores on EAT–26 compared to patients without disordered eating (after controlling for age, sex and BMI the difference remained significant).

*Abbreviations. AMDP, Manual for the Assessment & Documentation of Psychopathology; AN, anorexia nervosa; APS, Attenuated Psychosis Syndrome; BABS, Brown Assessment of Beliefs Scale; BED, binge eating disorder; BMI, Body Mass Index; BN, bulimia nervosa; BSABS, Bonn Scale for the Assessment of Basic Symptoms; DIGS, Diagnostic Interview for Genetic Studies; DSM-III-R, Diagnostic and Statistical Manual of Mental Disorders, Third Edition Revised; DSM-IV, Diagnostic and Statistical Manual of Mental Disorders, Fourth Edition; DSM-IV-TR, Diagnostic and Statistical Manual of Mental Disorders, fourth edition, text revision; DSM-5, Diagnostic and Statistical Manual of Mental Disorders, Fifth Edition; EAT, Eating Attitudes Test; EAT-26, Eating Attitude Test-26; ED, eating disorders; EDNOS, eating disorders not otherwise specified; K-SADS-PL, Schedule for Affective Disorders and Schizophrenia for School-Age Children-Present and Lifetime vision; MA, meta-analysis; MINI, Mini International Neuropsychiatric Interview; NEQ, Night Eating Questionnaire; NES, Night Eating Syndrome; NR, not reported; PANSS, Positive and Negative Symptom Scale; PDI, Peters et al. Delusions Inventory; PSYRATS, Psychotic Symptom Rating Scales-Delusions; SANS, Scale for the Assessment of Negative Symptoms; SAPS, Scale for the Assessment of Positive Symptoms; SCID, Structured Clinical Interview for DSM Disorders; SCID-I, Structured Clinical Interview for DSM-IV Axis I Disorders; SCID-I/P Version 2.0, Structured Interview for DSM-IV Axis I Disorders – Patient Edition; SCID-IV, Clinical interview using DSM-IV criteria; SD, Standard Deviation; SIPS, Structured Interview for Psychosis-Risk Syndromes; SOPS, Scale of Prodromal Symptoms; SSD, schizophrenia spectrum disorders; SZ, patients with schizophrenia.*

The selected articles represent 40 unique studies published from 1984 to 2024. Sixteen articles contain data on clinical populations with EDs, 12 on clinical populations with psychosis, three studies compared data from clinical populations with EDs and psychotic disorders, and 12 articles utilised general population samples. The three comparative studies with data on EDs and psychosis were grouped with the ED studies. Study characteristics are presented in Tables 1, 2 and 3. The risk of bias and the methodological quality of the selected studies are presented in Appendix E. All studies were of high or moderate quality.

### Narrative synthesis: general population studies

In total 12 articles with community populations were included in this review, which represented 10 unique studies, as three population cohort retrospective studies in Sweden and Denmark presented data of the same population (Plana-Ripoll et al., 2019; Zhang et al., 2021, 2023). The publication year of the studies ranged from 2016 to 2024. The studies were conducted in European countries (three in the United Kingdom, one in Sweden and Denmark), Africa (one in Kenya, one in Tunisia), Middle East (one in Iran), USA (one) and Australia (one). One study contained data from 18 countries across all the continents. Six studies were cross-sectional studies, three were population-based cohort retrospective studies, two had prospective designs and one study had a retrospective design. Risk of bias scores ranged from 5 to 8 with nine studies rated as high quality and three as moderate quality.

An association between eating pathology and psychotic symptoms was identified in all but one study, by Mohammadi et al. (2020). Both cross-sectional and retrospective cohort studies demonstrated a strong association between EDs and schizophrenia spectrum disorders in adults and children. Up to 3.7% of children aged 9–10 years presented EDs with psychotic symptoms and 0.8% were currently diagnosed with co-occurring EDs and psychotic disorders (Convertino & Blashill, 2022). In a cross-sectional survey, 16.4% to 24.7% of adults who reported psychotic-like experience had signs of EDs (Koyanagi, Stickley, & Haro, 2016). Individuals with AN or other EDs were at greater risk of co-occurring psychotic disorder (Rodgers, Marwaha, & Humpston, 2022) or developing schizophrenia or other psychotic disorders in the future (Zhang et al., 2021). However, Zhang et al.’s (2021) study was the only investigation in our review to identify ED diagnosis preceding a schizophrenia diagnosis.

By contrast, people with premorbid psychotic-like experiences were at greater risk of eating pathology (McGrath et al., 2016; Solmi, Melamed, Lewis, & Kirkbride, 2018). Of note, 29% of children with psychotic like experiences at age 13 reported disordered eating behaviours by the age of 18 (Solmi et al., 2018) whilst McGrath et al. (2016) found that psychotic experiences were associated with subsequent onset of both bulimia nervosa and binge eating disorder. One longitudinal study demonstrated that the association between psychotic experiences and disordered eating twelve months later in high school students was mediated by body satisfaction and weight preoccupations (Fekih-Romdhane, Houissa, Cheour, Hallit, & Loch, 2024). Symptoms of paranoia were associated with body and weight concerns (Malcolm et al., 2022), while uncontrolled eating was associated with auditory hallucinations and other psychotic experiences (Koyanagi et al., 2016). Psychosis and binge eating disorder were strongly correlated (Mutiso et al., 2022).

### Narrative synthesis: eating disorders studies

Nineteen studies with ED clinical populations were included in this review. Three of these studies contained data both on clinical populations with ED and psychosis (Camprodon-Boadas et al., 2023; Lysaker et al., 2023; Mountjoy, Farhall, & Rossell, 2014). They represent 18 unique studies and two papers reported on the same dataset (De Young et al., 2022; Kambanis et al., 2023). Seven studies explored the delusionality of the dominant beliefs of patients with ED and twelve studies explored the association of eating pathology with psychosis or psychotic symptoms. They were published from 1984 to 2023. Nine studies were conducted in USA, five in Italy, two in Greece, one in Spain, one in Australia and one in Russia. Ten studies were cross-sectional studies, seven were case-control studies and two were prospective observational designs. Thirteen studies were of high methodological quality according to Newcastle-Ottawa Scale criteria – receiving a total score of 8 or 7. Six studies were of medium quality and were rated as 6–5.

Paranoia was associated with disordered eating symptoms (Catone, Salerno, Muzzo, Lanzara, & Gritti, 2021) and patients with AN reported more psychotic symptoms such as paranoia (Pruccoli, Chiavarino, Nanni, & Parmeggiani, 2023) compared to patients with bulimia nervosa (Lysaker et al., 2023). The most common dominant beliefs in patients with EDs were related to food, body shape, weight fear and fear of losing control (Kambanis et al., 2023; Steinglass, Eisen, Attia, Mayer, & Walsh, 2007). Likewise, 10%–28.2% patients with ED were classified as having delusional body image beliefs according to the Brown Assessment of Beliefs Scale (BABS) (De Young et al., 2022; Kambanis et al., 2023; Konstantakopoulos et al., 2012; Konstantakopoulos, Ioannidi, Patrikelis, & Gonidakis, 2020; Mountjoy, 2014), 30.4% had overvalued ideas (Konstantakopoulos et al., 2020) and 28%–34% had poor insight (De Young et al., 2022; Kambanis et al., 2023; Mountjoy, 2014; Steinglass et al., 2007). Delusional beliefs were significantly more frequent in patients with AN compared to patients with bulimia nervosa and controls (Konstantakopoulos et al., 2012, 2020). The preoccupation and distress in relation to the dominant belief was higher in patients with AN compared to patients with schizophrenia (Mountjoy, 2014). A comparative study between patients with AN, first-episode of psychosis and OCD demonstrated that patients with first-psychosis episode and patients with AN reported higher conviction of their belief and delusionality compared to patients with OCD (Camprodon-Boadas et al., 2023). Patients with delusional beliefs and poor insight reported more disordered eating symptoms (Konstantakopoulos et al., 2012), more psychotic symptoms (Steinglass et al., 2007) more body-checking behaviours, purgative behaviours, restricting eating and fear of fatness (De Young et al., 2022; Kambanis et al., 2023).

### Narrative synthesis: psychotic disorders studies

Twelve articles and unique studies that presented data on clinical populations with psychotic disorders were included, of which seven were cross-sectional studies, four case-control, and one prospective observational study. The studies were published between 1985 and 2023. Most of the studies were conducted in the USA (four studies), Middle East (two in Turkey, one in Iran, one in Israel) and in European countries (one in Switzerland, one in Greece). One study was conducted in Asia (Singapore) and one in Africa (Egypt). Risk of bias scores ranged from 5 to 8, with 11 studies of high quality and one of moderate quality.

The findings of the studies in patients with psychosis demonstrated a strong association between psychotic disorders and eating pathology. Patients with psychosis experienced more EDs and disordered eating symptoms compared to controls (Khazaal, Frésard, Borgeat, & Zullino, 2006; Khosravi, 2020; Lyketsos et al., 1985), with 11.3% to 41.5% of patients with psychosis reporting disordered eating symptomatology (Fawzi & Fawzi, 2012; Khosravi, 2020; Stein, Zemishlani, Shahal, & Barak, 2005; Teh et al., 2021). Patients with disordered eating behaviours reported more and greater psychotic symptoms (Fawzi & Fawzi, 2012; Malaspina et al., 2019; Stein et al., 2005).

### Meta–analysis

**Figure 2.**
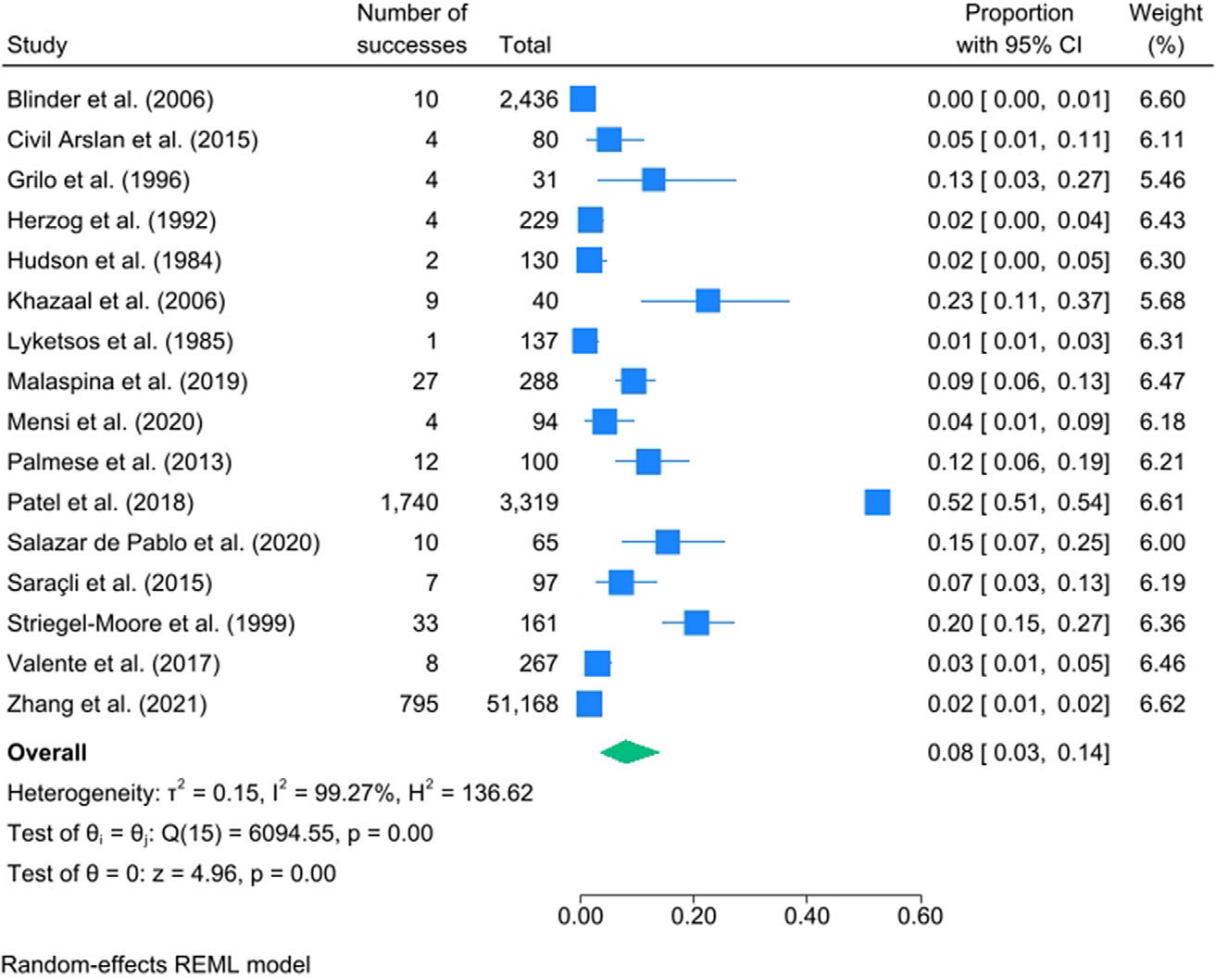
Meta-analysis of comorbidity across eating disorders and psychotic disorders.

Sixteen studies met inclusion criteria for meta-analysis of the comorbidity between EDs and psychotic disorders (see Figure 2). The mean proportion of comorbidity expressed as a percentage was 8% [95% CI: 3, 14], with high levels of heterogeneity [I^2^ = 99.27%].

### Subgroup analysis – Clinical populations with eating disorders

The mean proportion of comorbidity was 8% [95% CI: 2, 19; I^2^ = 99.13%] across nine studies with clinical populations with EDs (see Appendix F).

### Subgroup analysis – Clinical populations with psychosis

The mean proportion of comorbidity was 9% [95% CI: 4, 15; I^2^ = 84.18%] across seven studies with clinical populations with psychosis (see Appendix G).

### Meta-analysis by region

Most studies were from North America or Europe. For North America, the mean proportion of comorbidity was 11% [95% CI: 3, 22; I^2^ = 99.09%] across nine studies; for Europe the proportion was 4% [95% CI: 0, 11; I^2^ = 95.16%] across five studies (see Appendices H and I respectively).

### Meta-analysis by study type

The mean proportion of comorbidity in cross-sectional studies was 9% [95% CI: 4, 17; I^2^ = 98.47%; *k* = 13] and in retrospective studies it was 3% [95% CI: 0, 9; I^2^ = 94.55%; *k* = 3] (see Appendices J and K).

### Publication bias

Inspection of funnel plots and Egger’s test results suggested no presence of publication bias except for the meta-analysis of European studies (*k* = 5; *p* value for Egger’s test = 0.03). Funnel plots with results for Egger’s tests are presented in Appendices L-R.

## Discussion

To our knowledge, this is the first systematic review exploring the association and comorbidity between eating pathology and psychosis across clinical and community populations. Our findings suggest a pooled comorbidity between EDs and psychotic disorders of 8% in our meta-analysis.

Our findings have important clinical and research implications. In terms of the development of EDs and psychosis, high levels of comorbidity could speak to shared aetiological pathways, potentially offering avenues for early intervention. For example, unusual experiences on the psychosis spectrum in early adolescence might represent a risk factor for eating pathology (Solmi et al., 2018), although these findings require replication in further samples. Future cohort studies could shed light on the onset and the prediction of psychosis and EDs through a detailed developmental framework or symptom-focused typology of symptoms associated with the intersections and/or similarities between EDs and psychotic disorders for adolescents. Earlier identification of risk factors for adolescents at higher risk of EDs and psychotic disorders, or increased risk of both due to the presence of symptoms of the other, could offer additional options for earlier intervention and improved treatment outcomes. In terms of EDs, psychological interventions that address experiences such as the ED voice at an early stage, before ED symptoms become entrenched, could help to prevent the onset and maintenance of enduring EDs. Moreover, given the relatively low rates of ED recovery (Solmi et al., 2024), addressing psychotic-symptoms such as delusions and the ED voice in patients with EDs could represent promising lines for future research. Additionally, low-intensity intervention at an early stage and early age in community children’s mental health services and schools, offering normalising psychoeducation and coping strategy enhancement to promote communication and help-seeking, and reduce symptom related distress and the internalisation of stigma, could improve longer-term outcomes. For patients with psychotic disorders, our findings suggest high rates of ED symptoms, particularly binge eating symptomology, which warrant clinical attention. Waite et al. (2022) identified appearance concerns as a theme in their study of patients with psychosis and their experience of weight gain in the context of antipsychotic treatment, which is a common phenomenon. Further qualitative research is needed to better understand the experiences of such patients, including the potential impact of changes in appetite and weight caused by antipsychotic medication and shifting self-perception through turbulent developmental periods on the development of ED symptoms.

### Limitations

An important factor to consider in interpreting our findings is the high degree of both statistical heterogeneity in the meta-analysis, as well as methodological heterogeneity overall. Whilst our meta-analysis only included studies which used either clinical diagnosis or formal interview assessments, studies ranged across a period of 40 years, in which time both diagnostic practice and manuals will have changed. Secondly, studies utilising self-report questionnaires and ‘cut-off scores’ to assess psychosis and eating disorder pathology will provide higher estimates of comorbidity as compared with interview-based assessment methods. Studies using active ascertainment methods will likewise identify higher rates of comorbidity than studies relying on retrospective methods such as registry studies (Uher et al., 2023). We were unable to conduct a meta-analysis of correlation due to inconsistent measurement and reporting, and we recommend standardised reporting of correlations using Pearson’s *r* in future studies to aid future meta-analyses.

A further limitation is the lack of validation studies of ED measures in psychosis populations, and psychosis measures in ED populations. Psychometric studies are required and should include qualitative studies to explore content validity, as well as quantitative studies of properties such as factor structure and concurrent validity. Finally, it should be noted that studies have predominantly been conducted in Western countries, with frequent non-reporting of the ethnicity of participants, thereby hampering generalisability. Further research is needed to understand the association between ED and psychosis in diverse populations, including in the Global South, with transparent reporting on social determinants.

## Conclusion

Psychotic disorders and EDs represent two serious psychiatric conditions with profound implications for patients, their families and society. Our review suggests that the two conditions are associated, and frequently co-occur in clinical populations. Data from community samples also identify their co-occurrence in childhood and adolescence (e.g. Convertino & Blashill, 2022; Solmi et al., 2018), highlighting the need for further research utilising large datasets to investigate the developmental pathways and early clinical indicators that may be involved in their onset to inform more holistic assessment approaches and preventative intervention options. Finally, our review highlights the potential for new intervention studies to address comorbid symptoms in clinical populations, especially those that may maintain the disorders. Qualitative research and co-design approaches are indicated to develop such interventions in collaboration with people with lived experience of co-occurring ED and psychosis. This approach would help expand knowledge beyond arbitrary diagnoses and treatment pathways, developing a rich and experience-led understanding of the intersections between ED and psychotic disorder onset, presentation, and maintenance to advance identification and treatment options.

## Supporting information

Drymonitou et al. supplementary material Drymonitou et al. supplementary material

## Supplementary material

To view supplementary material for this article, please visit http://doi.org/10.1017/S003329172500114X.
