# Supplementary material for: The association between disordered eating and psychosis in clinical and non-clinical populations: a systematic review and meta-analysis
Source: Psychol Med. 2025 May 28;55:e160. doi: 10.1017/S003329172500114X (PMC12150339; doi:10.1017/S003329172500114X)

**Appendices: The association between eating disorders and psychosis in clinical and non-clinical populations: a systematic review**

**Table of contents**

[**Appendix A: PRISMA Checklist** 3](#_Toc198237202)

[**Appendix B: Search strategy and eligibility criteria** 6](#_Toc198237203)

[**i.** **Search strategy** 6](#_Toc198237204)

[**ii.** **Validated measurements for psychosis and eating pathology** 9](#_Toc198237205)

[**iii.** **Diagnostic criteria for eating disorders and psychotic disorders** 11](#_Toc198237206)

[**Appendix C: references of included studies** 19](#_Toc198237207)

[**Appendix D: Excluded studies at full text screening** 27](#_Toc198237208)

[**Appendix F: *Forest plot showing comorbidity of eating disorders and psychotic disorders in clinical samples with eating disorders*** 43](#_Toc198237209)

[**Appendix G: *Forest plot showing comorbidity of eating disorders and psychotic disorders in clinical samples with psychosis*** 43](#_Toc198237210)

[**Appendix H: Forest plot for meta-analysis of the comorbidity between eating disorders and psychotic disorders across North American studies** 44](#_Toc198237211)

[**Appendix I: Forest plot for meta-analysis of the comorbidity between eating disorders and psychotic disorders across European studies** 44](#_Toc198237212)

[**Appendix J: Forest plot for meta-analysis of the comorbidity between eating disorders and psychotic disorders across cross-sectional studies** 45](#_Toc198237213)

[**Appendix K: Forest plot for meta-analysis of the comorbidity between eating disorders and psychotic disorders across retrospective studies** 45](#_Toc198237214)

[**Appendix L: Funnel plot for meta-analysis of the comorbidity between eating disorders and psychotic disorders across clinical and general population samples (*k* = 16; *p* value for Egger’s test = 0.58)** 46](#_Toc198237215)

[**Appendix M: Funnel plot for meta-analysis of the comorbidity between eating disorders and psychotic disorders across clinical populations with eating disorders (*k* = 9; *p* value for Egger’s test = 0.76)** 47](#_Toc198237216)

[**Appendix N: Funnel plot for meta-analysis of the comorbidity between eating disorders and psychotic disorders across clinical populations with psychosis (k = 7; *p* value for Egger’s test = 0.12)** 48](#_Toc198237217)

[**Appendix O: Funnel plot for meta-analysis of the comorbidity between eating disorders and psychotic disorders across North American studies (*k* = 9; *p* value for Egger’s test = 0.90)** 49](#_Toc198237218)

[**Appendix P: Funnel plot for meta-analysis of the comorbidity between eating disorders and psychotic disorders across European studies (*k* = 5; *p* value for Egger’s test = 0.03)** 50](#_Toc198237219)

[**Appendix Q: Funnel plot for meta-analysis of the comorbidity between eating disorders and psychotic disorders across cross-sectional studies (*k* = 13; *p* value for Egger’s test = 0.88)** 51](#_Toc198237220)

[**Appendix R: Funnel plot for meta-analysis of the comorbidity between eating disorders and psychotic disorders across retrospective studies (k = 3; *p* value for Egger’s test = 0.79)** 52](#_Toc198237221)

# **Appendix A: PRISMA Checklist**

| **Section and Topic** | **Item #** | **Checklist item** | **Location where item is reported** |
| --- | --- | --- | --- |
| **TITLE** | | |  |
| Title | 1 | Identify the report as a systematic review. | Title page |
| **ABSTRACT** | | |  |
| Abstract | 2 | See the PRISMA 2020 for Abstracts checklist. | 1 |
| **INTRODUCTION** | | |  |
| Rationale | 3 | Describe the rationale for the review in the context of existing knowledge. | 2-5 |
| Objectives | 4 | Provide an explicit statement of the objective(s) or question(s) the review addresses. | 5 |
| **METHODS** | | |  |
| Eligibility criteria | 5 | Specify the inclusion and exclusion criteria for the review and how studies were grouped for the syntheses. | 6-7 |
| Information sources | 6 | Specify all databases, registers, websites, organisations, reference lists and other sources searched or consulted to identify studies. Specify the date when each source was last searched or consulted. | 5, S5-S7 |
| Search strategy | 7 | Present the full search strategies for all databases, registers and websites, including any filters and limits used. | 8, S5-S7 |
| Selection process | 8 | Specify the methods used to decide whether a study met the inclusion criteria of the review, including how many reviewers screened each record and each report retrieved, whether they worked independently, and if applicable, details of automation tools used in the process. | 6, S5,S8-S17 |
| Data collection process | 9 | Specify the methods used to collect data from reports, including how many reviewers collected data from each report, whether they worked independently, any processes for obtaining or confirming data from study investigators, and if applicable, details of automation tools used in the process. | 5-7, S5 |
| Data items | 10a | List and define all outcomes for which data were sought. Specify whether all results that were compatible with each outcome domain in each study were sought (e.g. for all measures, time points, analyses), and if not, the methods used to decide which results to collect. | 6, S5 |
|  | 10b | List and define all other variables for which data were sought (e.g. participant and intervention characteristics, funding sources). Describe any assumptions made about any missing or unclear information. | 6 |
| Study risk of bias assessment | 11 | Specify the methods used to assess risk of bias in the included studies, including details of the tool(s) used, how many reviewers assessed each study and whether they worked independently, and if applicable, details of automation tools used in the process. | 6-7 |
| Effect measures | 12 | Specify for each outcome the effect measure(s) (e.g. risk ratio, mean difference) used in the synthesis or presentation of results. | 6-7 |
| Synthesis methods | 13a | Describe the processes used to decide which studies were eligible for each synthesis (e.g. tabulating the study intervention characteristics and comparing against the planned groups for each synthesis (item #5)). | 7 |
|  | 13b | Describe any methods required to prepare the data for presentation or synthesis, such as handling of missing summary statistics, or data conversions. | 7 |
|  | 13c | Describe any methods used to tabulate or visually display results of individual studies and syntheses. | 7 |
|  | 13d | Describe any methods used to synthesize results and provide a rationale for the choice(s). If meta-analysis was performed, describe the model(s), method(s) to identify the presence and extent of statistical heterogeneity, and software package(s) used. | 7 |
|  | 13e | Describe any methods used to explore possible causes of heterogeneity among study results (e.g. subgroup analysis, meta-regression). |  |
|  | 13f | Describe any sensitivity analyses conducted to assess robustness of the synthesized results. | 7 |
| Reporting bias assessment | 14 | Describe any methods used to assess risk of bias due to missing results in a synthesis (arising from reporting biases). | 6 |
| Certainty assessment | 15 | Describe any methods used to assess certainty (or confidence) in the body of evidence for an outcome. | 7 |
| **RESULTS** | | |  |
| Study selection | 16a | Describe the results of the search and selection process, from the number of records identified in the search to the number of studies included in the review, ideally using a flow diagram. | 8,9 |
|  | 16b | Cite studies that might appear to meet the inclusion criteria, but which were excluded, and explain why they were excluded. | 7, S26-S38 |
| Study characteristics | 17 | Cite each included study and present its characteristics. | 8-40, S18-S25 |
| Risk of bias in studies | 18 | Present assessments of risk of bias for each included study. | S39-S41 |
| Results of individual studies | 19 | For all outcomes, present, for each study: (a) summary statistics for each group (where appropriate) and (b) an effect estimate and its precision (e.g. confidence/credible interval), ideally using structured tables or plots. | 11-17, 20-32, 34-40 |
| Results of syntheses | 20a | For each synthesis, briefly summarise the characteristics and risk of bias among contributing studies. | 8-10, 18-19, 33 |
|  | 20b | Present results of all statistical syntheses conducted. If meta-analysis was done, present for each the summary estimate and its precision (e.g. confidence/credible interval) and measures of statistical heterogeneity. If comparing groups, describe the direction of the effect. | 11-12 |
|  | 20c | Present results of all investigations of possible causes of heterogeneity among study results. | 12 |
|  | 20d | Present results of all sensitivity analyses conducted to assess the robustness of the synthesized results. | - |
| Reporting biases | 21 | Present assessments of risk of bias due to missing results (arising from reporting biases) for each synthesis assessed. | S39-S41 |
| Certainty of evidence | 22 | Present assessments of certainty (or confidence) in the body of evidence for each outcome assessed. | 11-12 |
| **DISCUSSION** | | |  |
| Discussion | 23a | Provide a general interpretation of the results in the context of other evidence. | 41, 42 |
|  | 23b | Discuss any limitations of the evidence included in the review. | 42 |
|  | 23c | Discuss any limitations of the review processes used. | 42 |
|  | 23d | Discuss implications of the results for practice, policy, and future research. | 41-43 |
| **OTHER INFORMATION** | | |  |
| Registration and protocol | 24a | Provide registration information for the review, including register name and registration number, or state that the review was not registered. | 5 |
|  | 24b | Indicate where the review protocol can be accessed, or state that a protocol was not prepared. | 5 |
|  | 24c | Describe and explain any amendments to information provided at registration or in the protocol. | - |
| Support | 25 | Describe sources of financial or non-financial support for the review, and the role of the funders or sponsors in the review. | Title page |
| Competing interests | 26 | Declare any competing interests of review authors. | Title page |
| Availability of data, code and other materials | 27 | Report which of the following are publicly available and where they can be found: template data collection forms; data extracted from included studies; data used for all analyses; analytic code; any other materials used in the review. | S1-S41 |

# **Appendix B: Search strategy and eligibility criteria**

## **Search strategy**

The electronic databases Web of Science (including Science Citation Index Expanded (SCI-EXPANDED) from 1900 to present; Social Sciences Citation Index (SSCI) from 1956 to present; Arts & Humanities Citation Index (A&HCI) from 1975 to present; Conference Proceedings Citation Index- Science (CPCI-S) from 1990 to present; Conference Proceedings Citation Index- Social Science & Humanities (CPCI-SSH) from 1990 to present; Emerging Sources Citation Index (ESCI) from 2015 to present), Medline and PsycINFO (accessed through Ovid) were searched from the 3^rd^ to 15^th^ of January 2021. The key search items “eating disorder* OR feeding disorder* OR purging* OR disordered eating OR bulimi* OR anorexi* OR binge*” in combination with “perceptual abnormalit* OR negative symptom* OR unusual experienc* OR unusual belief* OR delusion* OR hallucinat* OR paranoi* OR grandio* OR schizo* OR psychos?s OR psychotic*” were used. The results of the databases were limited to articles, English language and studies conducted on humans (See Supplementary Table 1 for details). There was no restriction on year of publication.

| **Supplementary Table 1**  *Database searches* | |
| --- | --- |
| **Database: PsycINFO (991 hits, 26/02/24)** | |
| **#1** | exp Eating Disorders/ OR anorexi*.mp. OR bulimi*.mp. OR binge eat*.mp. OR eating disorder*.mp OR exp Feeding Disorders/ OR exp Anorexia Nervosa/ OR exp Bulimia/ OR exp Binge Eating/ OR avoidant restrictive food intake disorder.mp. OR ARFID.mp. OR OSFED.mp. OR (other specified feeding or eating disorder*).mp. OR pica.mp. OR rumination disorder*.mp. OR (unspecified feeding or eating disorder*).mp. OR purging disorder*.mp OR night eating syndrome*.mp. OR EDNOS.mp. OR (eating disorder not otherwise specified).mp. |
| **#2** | perceptual abnormalit* OR negative symptom* OR unusual experienc* OR unusual belief* OR delusion* OR hallucinat* OR paranoi* OR grandio* OR schizo* OR psychos?s OR psychotic* |
| **#3** | 1 and 2 |
| **Filters** | limit 3 to (("0300 clinical trial" or "0400 empirical study" or "0430 followup study" or "0450 longitudinal study" or "0451 prospective study" or "0453 retrospective study" or 1800 quantitative study) and (100 childhood <birth to age 12 yrs> or 200 adolescence <age 13 to 17 yrs> or "300 adulthood <age 18 yrs and older>" or 320 young adulthood <age 18 to 29 yrs> or 340 thirties <age 30 to 39 yrs> or 360 middle age <age 40 to 64 yrs> or "380 aged <age 65 yrs and older>" or "390 very old <age 85 yrs and older>") and ("0100 journal" or "0110 peer-reviewed journal") and journal article and english and (human or male or female or inpatient or outpatient)) |
| **Database: Medline (1016 hits, 26/02/24)** | |
| **#1** | exp "Feeding and Eating Disorders"/eating disorder* OR anorexi*.mp. OR bulimi*.mp. OR binge eat*.mp. OR eating disorder*.mp. OR exp Anorexia Nervosa/ OR exp Bulimia Nervosa/ or exp Bulimia/ OR exp Binge-Eating Disorder/ OR exp "Feeding and Eating Disorders of Childhood"/ or exp "Feeding and Eating Disorders"/ or exp Avoidant Restrictive Food Intake Disorder/ OR ARFID.mp. OR OSFED.mp. OR (other specified feeding or eating disorder*).mp. OR pica.mp. or exp Pica/ OR rumination disorder*.mp. OR (unspecified feeding or eating disorder*).mp. OR purging disorder*.mp OR night eating syndrome*.mp. OR EDNOS.mp. OR (eating disorder not otherwise specified).mp. |
| **#2** | perceptual abnormalit* OR negative symptom* OR unusual experienc* OR unusual belief* OR delusion* OR hallucinat* OR paranoi* OR grandio* OR schizo* OR psychos?s OR psychotic* |
| **#3** | 1 and 2 |
| **Filters** | limit 3 to (("all child (0 to 18 years)" or "all adult (19 plus years)" or "adolescent (13 to 18 years)" or "young adult (19 to 24 years)" or "adult (19 to 44 years)" or "young adult and adult (19-24 and 19-44)" or "middle age (45 to 64 years)" or "middle aged (45 plus years)" or "all aged (65 and over)" or "aged (80 and over)") and english and (clinical study or clinical trial, all or journal article or observational study or randomized controlled trial) and "humans only (removes records about animals)") |
| **Database: Web of science (3538 hits, 26/02/24)** | |
| **#1** | TS=(anorexi* OR "eating disorder*" OR bulimi* OR "binge eat*" OR "feeding disorder*" OR "anorexia nervosa" OR bulimia OR "binge eating disorder*" OR "avoidant restrictive food intake disorder*" OR ARFID OR "Avoidant Restrictive Food Intake Disorder*" OR OSFED OR "other specified feeding or eating disorder*" OR pica OR "rumination disorder*" OR "unspecified feeding or eating disorder*" OR "purging disorder*" OR "night eating syndrome*" OR EDNOS OR "eating disorder not otherwise specified" ) |
| **#2** | TS=(perceptual abnormalit* OR negative symptom* OR unusual experienc* OR unusual belief* OR delusion* OR hallucinat* OR paranoi* OR grandio* OR schizo* OR psychos?s OR psychotic*) |
| **#3** | #1 AND #2 |
| **Filters** | limit 3 to English language and articles |

## **Validated measurements for psychosis and eating pathology**

| **Supplementary Table 2**  *Validated measurements for psychosis and psychotic symptoms* | |
| --- | --- |
| A. The following instruments are considered to define the UHR state ^a^ | Comprehensive Assessment of At-Risk Mental States (CAARMS),    Structured Interview for Psychosis-risk Syndromes (SIPS),  Early Recognition Inventory (ERIraos). |
| B. The following instruments are considered for full-flown psychotic symptoms | Positive and Negative Syndrome Scale (PANSS),  Scale for the Assessment of Negative Symptoms (SANS),  Scale for the Assessment of Positive Symptoms (SAPS). |
| C. The following instruments help evaluate a broad range of psychological problems and symptoms ^b^ | Brief Psychiatric Rating Scale (BPRS7),  Symptom Checklist-90-Revised (SCL-90-R) |
| D. The following instruments are considered to define basic psychotic symptoms | Bonn Scale for the Assessment of Basic Symptoms (BSABS),  Basel Screening Instrument for Psychosis (BSIP),  Schizophrenia Proneness Instrument- Adult (SPI-A) and Child and Youth (SPI-CY) version. |
| *Notes.*  ^a^ Instruments are used to evaluate attenuated/subthreshold symptoms and brief limited symptoms etc (CHR-P criteria), not psychosis.  ^b^ Instruments used are used as screening scales  Abbreviations: UHR, Ultra high risk for psychosis; CHR-P, Clinical high risk for psychosis | |

| **Supplementary Table 3**  *Validated measurements for eating pathology* | |
| --- | --- |
| A. Questionnaires used to evaluate disordered eating attitudes and behaviours | Eating Attitudes Test (EAT),  Eating Disorder Inventory (EDI),  Eating Disorder Examination Questionnaire (EDE-Q),  Body Attitude test (BAT),  Bulimia Criteria Questionnaire,  Binge Eating Scale,  Bulimic Investigatory Test Edinburgh (BITE). |
| B. Clinical interviews used for diagnosis of eating disorders | Eating Disorder Examination (EDE) |
| C. Clinical interviews used for evaluation of a broad range psychological symptoms | Structured Clinical Interview for DSM-IV Axis I Disorders (SCD-I) |
|  | |

## **Diagnostic criteria for eating disorders and psychotic disorders**

| **Supplementary Table 4**  *Clinical criteria for feeding and eating disorders according to DSM-V* | | |  |
| --- | --- | --- | --- |
| **Diagnoses** | | **Diagnostic criteria according to DSM-V** |  |
| Anorexia nervosa | | 1. Persistent dietary restriction leading to significant low body weight or failure to meet/maintain minimally expected body weight according to individual’s age, gender and developmental stage 2. Intense fear of gaining weight or becoming fat despite low body weight, or persistent preoccupation with behaviours related to weight gain 3. Distorted image of one’s body weight or shape   Anorexia nervosa can be classified as:   - restring subtype when weight loss is accomplished mainly through diet, fasting or excessive exercise, while individual refrained from engaging in binge-eating and purging behaviours during the last three months; - binge-eating/purging subtype when individual engaged in recurrent bingeing and purging episodes during the last three months, including self-induced vomiting or misuse of laxatives, enemas or diuretics.   Severity is based for adults on one’s current body mass index (BMI):   - Mild: BMI ≥ 17 kg/m^2^ - Moderate: BMI 16-16.99 kg/m^2^ - Severe: BMI 15-15.99 kg/m^2^ - Extreme: BMI < 15 kg/m^2^ |  |
| Bulimia nervosa | | 1. Recurrent episodes of binge eating which are characterised by: 2. Eating in a discrete period of time an amount of food that is larger than what most individuals would eat in a similar period and under similar conditions 3. A sense of lack of control over eating during the episode (such as that one cannot stop eating or control the kind and amount of food that is being consumed) 4. Recurrent inappropriate compensatory behaviours aiming at preventing weight gain (such as self-induced vomiting, misuse of diuretics, laxatives, fasting and excessive exercise) 5. Binge eating and compensatory behaviours should occur for at least once a week for 3 months 6. Self-evaluation is remarkedly related to by body shape and weight 7. Eating disturbance does not occur during episodes of anorexia nervosa |  |
|  | |  |  |
| **Supplementary Table 4**  *Clinical criteria for feeding and eating disorders according to DSM-V (continued)* | | | |
| **Diagnoses** | | **Diagnostic criteria according to DSM-V** | |
| Binge eating disorder | | 1. Recurrent episodes of binge eating which are characterised by: 2. Eating in a discrete period of time an amount of food that is larger than what most individuals would eat in a similar period and under similar conditions 3. A sense of lack of control over eating during the episode (such as that one cannot stop eating or control the kind and amount of food that is being consumed) 4. Binge eating episodes are related to three or more of the below: 5. Eating much more rapidly than normal 6. Eating until feeling uncomfortably full 7. Eating large amounts of food when not feeling physically hungry 8. Eating alone due to feeling embarrassed of how much one is eating 9. Feeling disgusted with oneself, guilty or depressed afterwards 10. Remarkable distress related to the presence of binge eating 11. Binge eating occurs at least once a week for 3 months 12. Binge eating is not related to recurrent use of inappropriate compensatory behaviours and does not occur during the course of bulimia or anorexia nervosa | |
| Avoidant/ restrictive food intake disorder | | 1. An eating or feeding disturbance characterised by persistent failure to meet the appropriate nutritional or energy needs, related to the following: 2. Significant weight loss or failure to achieve expected weight gain 3. Significant nutritional deficiency 4. Dependence on oral nutritional supplements or enteral feeding 5. Coupled with deficiency in psychosocial functioning 6. It is not explained by lack of food availability or relevant cultural sanctioned practices 7. Eating disturbance does not occur during the course of anorexia or bulimia nervosa and there is no evidence of distorted body weight or shape image 8. Eating disturbances are not attributed to concurrent medical conditions or other mental disorders. | |
| Other specified or eating disorder | | - Atypical anorexia nervosa: although all the criteria for anorexia nervosa are met, individual’s weight is within or above normal range - Bulimia nervosa (of low frequency/limited edition): binge eating, and compensatory behaviours occur less than once a week or for less than 3 months - Binge eating disorder (of low frequency/limited edition): binge eating occurs less than once a week or for less than 3 months - Purging disorder: Recurrent purging behaviours aiming at altering weight or shape in the absence of binge eating - Night eating syndrome: Recurrent episodes of night eating, characterised by eating after awakening from sleep or excessive food consumption after evening meal | |
| *Note.* Studies with individuals who meet diagnostic criteria for pica and rumination disorder are excluded. | | | |

| **Supplementary Table 5**  *Clinical criteria for feeding or eating disorders according to ICD-11* | |
| --- | --- |
| **Diagnoses** | **Diagnostic criteria according to ICD-11** |
| Anorexia nervosa | *“Anorexia nervosa is characterised by significantly low body weight for the individual’s height, age and developmental stage that is not due to another health condition or to the unavailability of food. A commonly used threshold is body mass index (BMI) less than 18.5 kg/m^2^ in adults and BMI-for-age under 5th percentile in children and adolescents. Rapid weight loss (e.g. more than 20% of total body weight within 6 months) may replace the low body weight guideline since other diagnostic requirements are met. Children and adolescents may exhibit failure to gain weight as expected based on the individual developmental trajectory rather than weight loss. Low body weight is accompanied by a persistent pattern of behaviours to prevent restoration of normal weight, which may include behaviours aimed at reducing energy intake (restricted eating), purging behaviours (e.g. self-induced vomiting, misuse of laxatives), and behaviours aimed at increasing energy expenditure (e.g. excessive exercise), typically associated with a fear of weight gain. Low body weight or shape is central to the person's self-evaluation or is inaccurately perceived to be normal or even excessive”.* |
| Bulimia nervosa | *“Bulimia nervosa is characterised by frequent, recurrent episodes of binge eating (e.g. once a week or more over a period of at least one month). A binge eating episode is a distinct period of time during which the individual experiences a subjective loss of control over eating, eating notably more or differently than usual, and feels unable to stop eating or limit the type or amount of food eaten. Binge eating is accompanied by repeated inappropriate compensatory behaviours aimed at preventing weight gain (e.g. self-induced vomiting, misuse of laxatives or enemas, strenuous exercise). The individual is preoccupied with body shape or weight, which strongly influences self-evaluation. There is marked distress about the pattern of binge eating and inappropriate compensatory behaviour or significant impairment in personal, family, social, educational, occupational or other important areas of functioning. The individual does not meet the diagnostic requirements of anorexia nervosa”.* |
| Binge eating disorder | *“Binge eating disorder is characterised by frequent, recurrent episodes of binge eating (e.g. once a week or more over a period of several months). A binge eating episode is a distinct period of time during which the individual experiences a subjective loss of control over eating, eating notably more or differently than usual, and feels unable to stop eating or limit the type or amount of food eaten. Binge eating is experienced as very distressing and is often accompanied by negative emotions such as guilt or disgust. However, binge eating episodes are not regularly followed by inappropriate compensatory behaviours aimed at preventing weight gain (e.g. self-induced vomiting, misuse of laxatives or enemas, strenuous exercise). There is marked distress about the pattern of binge eating or significant impairment in personal, family, social, educational, occupational or other important areas of functioning”.* |
| **Supplementary Table 5**  *Clinical criteria for feeding or eating disorders according to ICD-11* | |
| **Diagnoses** | **Diagnostic criteria according to ICD-11** |
| Avoidant restrictive food intake disorder | *“Avoidant-restrictive food intake disorder (ARFID) is characterised by avoidance or restriction of food intake that results in: the intake of an insufficient quantity or variety of food to meet adequate energy or nutritional requirements that has resulted in significant weight loss, clinically significant nutritional deficiencies, dependence on oral nutritional supplements or tube feeding, or has otherwise negatively affected the physical health of the individual; or significant impairment in personal, family, social, educational, occupational or other important areas of functioning (for example due to avoidance or distress related to participating in social experiences involving eating). The pattern of eating behaviour is not motivated by preoccupation with body weight or shape. Restricted food intake and its effects on weight, other aspects of health, or functioning is not due to unavailability of food, not a manifestation of another medical condition (such as food allergies, hyperthyroidism) or mental disorder, and are not due to the effect of a substance or medication on the central nervous system including withdrawal effects”.* |
| Eating disorder not elsewhere classified | This category is used for individuals who do not meet all the diagnostic criteria for the above eating disorders |
| *Note.* Studies with individuals with a diagnosis of pica and rumination-regurgitation disorder are excluded. | |

| **Supplementary Table 6**  *Clinical criteria for schizophrenia spectrum and other psychotic disorders according to DSM-V* | |
| --- | --- |
| **Diagnoses** | **Diagnostic criteria according to DSM-V** |
| Schizophrenia | 1. Two or more of the following, must be present during a 1-month period (1, 2 or 3 must be present): 2. Delusions 3. Hallucinations 4. Disorganised speech 5. Grossly disorganised or catatonic behaviour 6. Negative symptoms (such as avolition, diminished emotional expression etc) 7. Diminished level of functioning in one or more major personal and interpersonal areas, since the onset of symptoms 8. An episode should last more than 6 months (symptoms at criterion A should be present at least for one month and may include periods with prodromal or residual symptoms) 9. Schizoaffective disorders and bipolar disorder or depression with psychotic features are ruled out 10. Symptoms are not attributed to physiological effects of a substance or other medical condition |
| Schizophreniform disorder | 1. Two or more of the following, must be present during a 1-month period (1, 2 or 3 must be present): 2. Delusions 3. Hallucinations 4. Disorganised speech 5. Grossly disorganised or catatonic behaviour 6. Negative symptoms (such as avolition, diminished emotional expression etc) 7. An episode should last more than 1 month and less than 6 months 8. Schizoaffective disorders and bipolar disorder or depression with psychotic features are ruled out 9. Symptoms are not attributed to physiological effects of a substance or other medical condition |
| Schizoaffective disorder | 1. An uninterrupted period of panic or depressive episode, concurrent with criterion A of schizophrenia 2. Presence of delusions or hallucinations for two or more weeks without major mood episodes 3. Presence of depressive or manic symptoms most of the time during the lifetime of the illness 4. Symptoms are not attributed to physiological effects of a substance or other medical conditions |
| Brief psychotic disorder | 1. Presence of one or more of the following symptoms (at least 1, 2 or 3 must be present): 2. Delusions 3. Hallucinations 4. Disorganised speech 5. Grossly disorganised or catatonic behaviour |
| **Supplementary Table 6**  *Clinical criteria for schizophrenia spectrum and other psychotic disorders according to DSM-V (continued)* | |
| **Diagnoses** | **Diagnostic criteria according to DSM-V** |
| Delusional disorder | 1. Presence of one or more delusions for a month or more 2. Criterion A of schizophrenia has never been met 3. General functioning is not markedly impaired 4. If major depressive or manic episodes occurred, their duration was briefer compared to delusional periods 5. Symptoms are not attributed to physiological effects of a substance or another medical condition and are not explained by another mental health (such as body dysmorphic disorder or obsessive-compulsive disorder)   Types of delusions: Erotomanic, Grandiose, Jealous, Persecutory, somatic type etc |
| Other specified schizophrenia spectrum and other psychotic disorder | 1. Persistent auditory hallucinations 2. Delusions with significant overlapping mood episodes 3. Attenuated psychosis syndrome |
| *Note.* Studies with individuals meeting criteria for substance/medication-induced psychotic disorder, psychotic disorder due to another medical condition and catatonia are excluded from our systematic review | |

| **Supplementary Table 7**  *Clinical criteria for schizophrenia spectrum and other primary psychotic disorders according to ICD-11* | |
| --- | --- |
| **Diagnoses** | **Diagnostic criteria according to ICD-11** |
| Schizophrenia | *Schizophrenia is characterised by significant impairments in reality testing and alterations in behaviour, including positive symptoms (such as persistent delusions, hallucinations, disorganised thinking, disorganised speech, grossly disorganised behaviour, and experiences of passivity and control), negative symptoms (such as blunted or flat affect, avolition) and psychomotor disturbances. The symptoms occur with sufficient frequency and intensity to deviate from expected cultural or subcultural norms. These symptoms are not attributed to another mental and behavioural disorder (such as mood disorders, delirium, or substance-induced).* |
| Schizoaffective disorder | *“Schizoaffective disorder is an episodic disorder in which the diagnostic requirements of schizophrenia and a manic, mixed, or moderate or severe depressive episode are met within the same episode of illness, either simultaneously or within a few days of each other. Prominent symptoms of schizophrenia (such as delusions, hallucinations, disorganisation in the form of thought, experiences of influence, passivity and control) are accompanied by typical symptoms of a moderate or severe depressive episode (including depressed mood, loss of interest, reduced energy), a manic episode (including extreme mood state characterised by euphoria, irritability, or expansiveness; increased activity or a subjective experience of increased energy) or a mixed episode. Psychomotor disturbances, including catatonia, may be present. Symptoms must have persisted for at least one month. The symptoms are not attributed to another medical condition (e.g. a brain tumour) or the effect of a substance or medication on the central nervous system (e.g. corticosteroids), including withdrawal (e.g. alcohol withdrawal)”.* |
| Schizotypal disorder | *“Schizotypal disorder is characterised by an enduring pattern (i.e. characteristic of the person’s functioning over a period of at least several years) of eccentricities in behaviour, appearance and speech, accompanied by cognitive and perceptual distortions, unusual beliefs, and discomfort with— and often reduced capacity for— interpersonal relationships. Symptoms may include constricted or inappropriate affect and anhedonia. Paranoid ideas, ideas of reference, or other psychotic symptoms, including hallucinations in any modality, may occur, but are not of sufficient intensity or duration to meet the diagnostic requirements of schizophrenia, schizoaffective disorder, or delusional disorder. The symptoms cause distress or impairment in personal, family, social, educational, occupational or other important areas of functioning”.* |
|  |  |
| **Supplementary Table 7**  *Clinical criteria for schizophrenia spectrum and other primary psychotic disorders according to ICD-11 (continued)* | |
| **Diagnoses** | **Diagnostic criteria according to ICD-11** |
| Acute and transient psychotic disorder | *“Acute and transient psychotic disorder is characterised by acute onset of psychotic symptoms that emerge without a prodrome and reach their maximal severity within two weeks. Symptoms may include delusions, hallucinations, disorganisation of thought processes, perplexity or confusion, and disturbances of affect and mood. Catatonia-like psychomotor disturbances may be present. Symptoms typically change rapidly, both in nature and intensity, from day to day, or even within a single day. The duration of the episode does not exceed 3 months, and most commonly lasts from a few days to 1 month. The symptoms are not a manifestation of another medical condition (e.g. a brain tumour) and are not due to the effect of a substance or medication on the central nervous system (e.g. corticosteroids), including withdrawal (e.g. alcohol withdrawal)”.* |
| Delusional disorder | *“Delusional disorder is characterised by the development of a delusion or set of related delusions, typically persisting for at least 3 months and often much longer, in the absence of a Depressive, Manic, or Mixed mood episode. The delusions are variable in content across individuals, but typically stable within individuals, although they may evolve over time. Other characteristic symptoms of Schizophrenia (such as clear and persistent hallucinations, negative symptoms, disorganised thinking, or experiences of influence, passivity, or control) are not present, although various forms of perceptual disturbances (including hallucinations, illusions, misidentifications of persons) thematically related to the delusion are still consistent with the diagnosis. Apart from actions and attitudes directly related to the delusion or delusional system, affect, speech, and behaviour are typically unaffected. The symptoms are not a manifestation of another medical condition (e.g., a brain tumour) and are not due to the effect of a substance or medication on the central nervous system (e.g. corticosteroids), including withdrawal effects (e.g. alcohol withdrawal)”.* |
| Unspecified non-organic psychosis | This category is used for non-organic psychosis with unknown aetiology |
| *Note.* Studies with individuals with a diagnosis of secondary psychotic syndrome, catatonia and substance-induced psychotic disorders are excluded. | |

# **Appendix C: references of included studies**

Blinder, B. J., Cumella, E. J., & Sanathara, V. A. (2006). Psychiatric Comorbidities of Female Inpatients With Eating Disorders. *Psychosomatic Medicine*, *68*(3), 454. https://doi.org/10.1097/01.psy.0000221254.77675.f5

Camprodon-Boadas, P., De la Serna, E., Plana, M. T., Flamarique, I., Lázaro, L., Borràs, R., Baeza, I., Tasa-Vinyals, E., Sugranyes, G., Ortiz, A. E., & Castro-Fornieles, J. (2023). Delusional beliefs in adolescents with anorexia nervosa, obsessive-compulsive disorder, or first-episode psychosis: A comparative study. *Psychiatry research, 328,* 115490. <https://doi.org/10.1016/j.psychres.2023.115490>

Catone, G., Salerno, F., Muzzo, G., Lanzara, V., & Gritti, A. (2021). Association between anorexia nervosa and other specified eating or feeding disorders and paranoia in adolescents: what factors are involved?. *Rivista di psichiatria, 56*(2), 100–106. https://doi.org/10.1708/3594.35768

Civil Arslan, F., Tiryaki, A., Aykut, D. S., Özkorumak, E., İlter, Z. Ç., & Günaydın, D. (2015). [The Prevalence of Night Eating Syndrome among Outpatient Overweight or Obese Individuals with Serious Mental Illness]. *Turk Psikiyatri Dergisi = Turkish Journal of Psychiatry*. <https://www.turkpsikiyatri.com/Data/UnpublishedArticles/yep9ac.pdf>

Convertino, A. D., & Blashill, A. J. (2022). Psychiatric comorbidity of eating disorders in children between the ages of 9 and 10. *Journal of child psychology and psychiatry, and allied disciplines*, *63*(5), 519–526. https://doi.org/10.1111/jcpp.13484

De Young, K., Bottera, A., Kambanis, E., Mancuso, C., Cass, K., Lohse, K., Benabe, J., Oakes, J., Watters, A., Johnson, C., & Mehler, P. (2022). Delusional intensity as a prognostic indicator among individuals with severe to extreme anorexia nervosa hospitalized at an acute medical stabilization program. *International Journal of Eating Disorders*, *55*(2), 215–222. https://doi.org/10.1002/eat.23641

Fawzi, M. H., & Fawzi, M. M. (2012). Disordered eating attitudes in Egyptian antipsychotic naive patients with schizophrenia. *Comprehensive Psychiatry*, *53*(3), 259–268. <https://doi.org/10.1016/j.comppsych.2011.04.064>

Fekih-Romdhane, F., Houissa, L., Cheour, M., Hallit, S., & Loch, A. A. (2024). Body image as a mediator in the relationship between psychotic experiences and later disordered eating: A 12-month longitudinal study in high school adolescents*. The International journal of social psychiatry, 70*(3), 518–530. <https://doi.org/10.1177/00207640231218686>

Grilo, C. M., Levy, K. N., Becker, D. F., Edell, W. S., & McGlashan, T. H. (1996). Comorbidity of DSM-III-R axis I and II disorders among female inpatients with eating disorders. *Psychiatric Services (Washington, D.C.)*, *47*(4), 426–429. <https://doi.org/10.1176/ps.47.4.426>

Herzog, D. B., Keller, M. B., Sacks, N. R., Yeh, C. J., & Lavori, P. W. (1992). Psychiatric Comorbidity in Treatment-Seeking Anorexics and Bulimics. *Journal of the American Academy of Child & Adolescent Psychiatry*, *31*(5), 810–818. https://doi.org/10.1097/00004583-199209000-00006

Hudson, J. I., Pope, H. G., & Jonas, J. M. (1984). Psychosis in Anorexia Nervosa and Bulimia. *The British Journal of Psychiatry*, *145*(4), 420–423. https://doi.org/10.1192/bjp.145.4.420.

Kambanis, P. E., Bottera, A. R., Mancuso, C. J., Cass, K., Lohse, K., Benabe, J., Oakes, J., Watters, A., Johnson, C., Mehler, P., & Young, K. P. D. (2023). Delusionality of beliefs among 50 adult females with severe and extreme anorexia nervosa upon admission to an acute medical stabilization facility. *Eating Disorders*, *31*(4), 353–361. https://doi.org/10.1080/10640266.2022.2135982

Khazaal, Y., Frésard, E., Borgeat, F., & Zullino, D. (2006). Binge eating symptomatology in overweight and obese patients with schizophrenia: A case control study. *Annals of General Psychiatry*, *5*(1), 15. https://doi.org/10.1186/1744-859X-5-15

Khosravi, M. (2020). Biopsychosocial factors associated with disordered eating behaviors in schizophrenia. *Annals of General Psychiatry*, *19*(1), 67. https://doi.org/10.1186/s12991-020-00314-2

Konstantakopoulos, G., Varsou, E., Dikeos, D., Ioannidi, N., Gonidakis, F., Papadimitriou, G., & Oulis, P. (2012). Delusionality of body image beliefs in eating disorders. *Psychiatry Research*, *200*(2), 482–488. https://doi.org/10.1016/j.psychres.2012.03.023

Konstantakopoulos, G., Ioannidi, N., Patrikelis, P., & Gonidakis, F. (2020). The impact of theory of mind and neurocognition on delusionality in anorexia nervosa. *Journal of Clinical and Experimental Neuropsychology*, *42*(6), 611–621. https://doi.org/10.1080/13803395.2020.1786504

Koyanagi, A., Stickley, A., & Haro, J. M. (2016). Psychotic-like experiences and disordered eating in the English general population. *Psychiatry Research*, *241*, 26–34. https://doi.org/10.1016/j.psychres.2016.04.045

Lyketsos, G. C., Paterakis, P., Beis, A., & Lyketsos, C. G. (1985). Eating Disorders in Schizophrenia. *The British Journal of Psychiatry*, *146*(3), 255–261. https://doi.org/10.1192/bjp.146.3.255

Lysaker, P. H., Chernov, N., Moiseeva, T., Sozinova, M., Dmitryeva, N., Makarova, A., Kukla, M., Myers, E., Karpenko, O., & Kostyuk, G. (2023). Contrasting Metacognitive, Emotion Recognition and Alexithymia Profiles in Bulimia, Anorexia, and Schizophrenia. *The Journal of Nervous and Mental Disease*, *211*(5), 348. https://doi.org/10.1097/NMD.0000000000001612

Malaspina, D., Walsh-Messinger, J., Brunner, A., Rahman, N., Corcoran, C., Kimhy, D., Goetz, R. R., & Goldman, S. B. (2019). Features of schizophrenia following premorbid eating disorders. *Psychiatry Research*, *278*, 275–280. https://doi.org/10.1016/j.psychres.2019.06.035

Malcolm, A., Phillipou, A., Neill, E., Rossell, S. L., & Toh, W. L. (2022). Relationships between paranoia and body image concern among community women. *Journal of Psychiatric Research*, *151*, 405–410. https://doi.org/10.1016/j.jpsychires.2022.05.007

McGrath, J. J., Saha, S., Al-Hamzawi, A., Andrade, L., Benjet, C., Bromet, E. J., Browne, M. O., Caldas de Almeida, J. M., Chiu, W. T., Demyttenaere, K., Fayyad, J., Florescu, S., de Girolamo, G., Gureje, O., Haro, J. M., ten Have, M., Hu, C., Kovess-Masfety, V., Lim, C. C. W., … Kessler, R. C. (2016). The Bidirectional Associations Between Psychotic Experiences and DSM-IV

Mensi, M. M., Rogantini, C., Nacinovich, R., Riva, A., Provenzi, L., Chiappedi, M., Balottin, U., & Borgatti, R. (2020). Clinical features of adolescents diagnosed with eating disorders and at risk for psychosis. *European Psychiatry*, *63*(1), e80. https://doi.org/10.1192/j.eurpsy.2020.80

Miotto, P., Pollini, B., Restaneo, A., Favaretto, G., Sisti, D., Rocchi, M. B. L., & Preti, A. (2010). Symptoms of psychosis in anorexia and bulimia nervosa. *Psychiatry Research*, *175*(3), 237–243. https://doi.org/10.1016/j.psychres.2009.03.011

Mohammadi, M. R., Mostafavi, S.-A., Hooshyari, Z., Khaleghi, A., Ahmadi, N., Molavi, P., Armani Kian, A., Safavi, P., Delpisheh, A., Talepasand, S., Hojjat, S. K., Pourdehghan, P., Ostovar, R., Hosseini, S. H., Mohammadzadeh, S., Salmanian, M., Alavi, S. S., Ahmadi, A., & Zarafshan, H. (2020). Prevalence, correlates and comorbidities of feeding and eating disorders in a nationally representative sample of Iranian children and adolescents. *International Journal of Eating Disorders*, *53*(3), 349–361. https://doi.org/10.1002/eat.23197

Mountjoy, R. L., F. Farhall, J., & L. Rossell, S. (2014). A phenomenological investigation of overvalued ideas and delusions in clinical and subclinical anorexia nervosa. *Psychiatry Research*, *220*(1), 507–512. https://doi.org/10.1016/j.psychres.2014.07.073

Mutiso, V. N., Ndetei, D. M., N Muia, E., K Alietsi, R., Onsinyo, L., Kameti, F., Masake, M., Musyimi, C., & Mamah, D. (2022). The prevalance of binge eating disorder and associated psychiatric and substance use disorders in a student population in Kenya – towards a public health approach. *BMC Psychiatry*, *22*(1), 122. https://doi.org/10.1186/s12888-022-03761-1

Palmese, L. B., Ratliff, J. C., Reutenauer, E. L., Tonizzo, K. M., Grilo, C. M., & Tek, C. (2013). Prevalence of night eating in obese individuals with schizophrenia and schizoaffective disorder. *Comprehensive Psychiatry*, *54*(3), 276–281. https://doi.org/10.1016/j.comppsych.2012.07.014

Patel, R. S., Olten, B., Patel, P., Shah, K., & Mansuri, Z. (2018). Hospitalization Outcomes and Comorbidities of Bulimia Nervosa: A Nationwide Inpatient Study. *Cureus*, *10*(5), e2583. https://doi.org/10.7759/cureus.2583

Plana-Ripoll, O., Pedersen, C. B., Holtz, Y., Benros, M. E., Dalsgaard, S., de Jonge, P., Fan, C. C., Degenhardt, L., Ganna, A., Greve, A. N., Gunn, J., Iburg, K. M., Kessing, L. V., Lee, B. K., Lim, C. C. W., Mors, O., Nordentoft, M., Prior, A., Roest, A. M., … McGrath, J. J. (2019). Exploring Comorbidity Within Mental Disorders Among a Danish National Population. *JAMA Psychiatry*, *76*(3), 259–270. https://doi.org/10.1001/jamapsychiatry.2018.3658

Pruccoli, J., Chiavarino, F., Nanni, C., & Parmeggiani, A. (2023). General psychopathological symptoms in children, adolescents, and young adults with anorexia nervosa—A naturalistic study on follow-up and treatment. *European Journal of Pediatrics*, *182*(3), 997–1007. https://doi.org/10.1007/s00431-022-04745-9

Rodgers, E., Marwaha, S., & Humpston, C. (2022). Co-occurring psychotic and eating disorders in England: Findings from the 2014 Adult Psychiatric Morbidity Survey. *Journal of Eating Disorders*, *10*(1), 150. https://doi.org/10.1186/s40337-022-00664-0

Salazar de Pablo, G., Guinart, D., Cornblatt, B. A., Auther, A. M., Carrión, R. E., Carbon, M., Jiménez-Fernández, S., Vernal, D. L., Walitza, S., Gerstenberg, M., Saba, R., Lo Cascio, N., Brandizzi, M., Arango, C., Moreno, C., Van Meter, A., Fusar-Poli, P., & Correll, C. U. (2020). DSM-5 Attenuated Psychosis Syndrome in Adolescents Hospitalized With Non-psychotic Psychiatric Disorders. *Frontiers in Psychiatry*, *11*. <https://doi.org/10.3389/fpsyt.2020.568982>

Salvatore, P., Baldessarini, R. J., Khalsa, H. K., & Tohen, M. (2021). Prodromal features in first-psychotic episodes of major affective and schizoaffective disorders. *Journal of Affective Disorders, 295,* 1251–1258. <https://doi.org/10.1016/j.jad.2021.08.099>

Saraçlı, Ö., Atasoy, N., Akdemir, A., Güriz, O., Konuk, N., Sevinçer, G. M., Ankaralı, H., & Atik, L. (2015). The prevalence and clinical features of the night eating syndrome in psychiatric out-patient population. *Comprehensive Psychiatry*, *57*, 79–84. https://doi.org/10.1016/j.comppsych.2014.11.007

Solmi, F., Melamed, D., Lewis, G., & Kirkbride, J. B. (2018). Longitudinal associations between psychotic experiences and disordered eating behaviours in adolescence: A UK population-based study. *The Lancet Child & Adolescent Health*, *2*(8), 591–599. https://doi.org/10.1016/S2352-4642(18)30180-9

Stein, D., Zemishlani, C., Shahal, B., & Barak, Y. (2005). Disordered eating in elderly female patients diagnosed with chronic schizophrenia*. The Israel journal of psychiatry and related sciences, 42*(3), 191–197.

Steinglass, J. E., Eisen, J. L., Attia, E., Mayer, L., & Walsh, B. T. (2007). Is Anorexia Nervosa a Delusional Disorder? An Assessment of Eating Beliefs in Anorexia Nervosa. *Journal of Psychiatric Practice®*, *13*(2), 65. <https://doi.org/10.1097/01.pra.0000265762.79753.88>

Striegel-Moore, R. H., Garvin, V., Dohm, F. A., & Rosenheck, R. A. (1999). Eating disorders in a national sample of hospitalized female and male veterans: detection rates and psychiatric comorbidity. *The International journal of eating disorders, 25*(4), 405–414. [https://doi.org/10.1002/(sici)1098-108x(199905)25:4<405::aid-eat5>3.0.co;2-f](https://doi.org/10.1002/(sici)1098-108x(199905)25:4%3c405::aid-eat5%3e3.0.co;2-f)

Teh, W. L., Mahesh, M. V., Abdin, E., Tan, J., Rahman, R. F. B. A., Satghare, P., Sim, K., Basu, S., Kandasami, G., Gupta, B., Chong, S. A., & Subramaniam, M. (2021). Negative affect moderates the link between body image dissatisfaction and disordered eating among psychiatric outpatients in a multi-ethnic Asian setting. *Singapore Medical Journal*, *62*(10), 535–541. <https://doi.org/10.11622/smedj.2020058>

Valente, S., Di Girolamo, G., Forlani, M., Biondini, A., Scudellari, P., De Ronchi, D., & Atti, A. R. (2017). Sex-specific issues in eating disorders: A clinical and psychopathological investigation. *Eating and Weight Disorders - Studies on Anorexia, Bulimia and Obesity*, *22*(4), 707–715. <https://doi.org/10.1007/s40519-017-0432-7>

Zhang, R., Kuja-Halkola, R., Birgegård, A., Larsson, H., Lichtenstein, P., Bulik, C. M., & Bergen, S. E. (2023). Association of family history of schizophrenia and clinical outcomes in individuals with eating disorders. *Psychological Medicine*, *53*(2), 371–378. <https://doi.org/10.1017/S0033291721001574>

Zhang, R., Larsen, J. T., Kuja-Halkola, R., Thornton, L., Yao, S., Larsson, H., Lichtenstein, P., Petersen, L. V., Bulik, C. M., & Bergen, S. E. (2021). Familial co-aggregation of schizophrenia and eating disorders in Sweden and Denmark. *Molecular Psychiatry*, *26*(9), 5389–5397. <https://doi.org/10.1038/s41380-020-0749-x>

# **Appendix D: Excluded studies at full text screening**

No validated measures for ED/ and psychosis reported (n = 34)

(Appolinario et al., 2022; Aragona et al., 2015; Ashour et al., 2022; Berthold et al., 2022; Brancati et al., 2022; Cao et al., 2022; de Beaurepaire, 2021; de Jonge et al., 2018; Flynn et al., 2023; Ganson et al., 2022; Hsu et al., 1981; Hubel et al., 2021; Kambanis et al., 2022; Kouidrat et al., 2018; Kurpad et al., 2010; Lasson & Raynal, 2021; Legg et al., 2023; Liu et al., 2021; Lu et al., 2022; McElroy et al., 2013; Mereu et al., 2022; Motteli et al., 2023; Muzi et al., 2021; Olgiati et al., 2022; Rasmussen et al., 2020; Regeneron Genetics Ctr et al., 2021; Risch et al., 2023; Sahlan & Sala, 2022; Sellbom et al., 2022; Sevillano-Jimenez et al., 2022; Small et al., 1981; Straface et al., 2023; Striegel-Moore, 1999; Yazdi et al., 2020).

Irrelevant outcome/study (n = 11)

(Diaz-Marsa et al., 2023; Johanson et al., 2022; Khazaal et al., 2010; Kim et al., 2023; Lee et al, 2022; Lee et al., 2021; Niwinski et al., 2021; Phillipou et al., 2021; Remberk et al., 2-21; Wainberg et al., 2022; Wojtaszek & Saules, n.d.)

ED and psychosis data not combined (n = 9)

(Buhren et al., 2024; Bunting et al., 2022; Eagles et al., 1990; Frigaard et al., 2023; John et al., 2022; Leutner et al., 2023; Lynham et al., 2022; Oswalt et al., 2020; Pedram et al., 2021).

Study on food addiction (n = 2)

(Horsager et al., 2022; Wattick et al., 2023).

No primary research (n = 1)

(Bou Khalil, 2021).

Qualitative study (n = 1)

(Ling et al,. 2022)

Review (n = 2)

(De Young & Rettler, 2023; Sankaranarayanan et al., 2021)

Genetic studies (n = 5)

(Ding et al., 2022; Kendler et al., 2021; Neumann et al., 2022; Polushina et al., 2021; Solmi et al., 2019).

Case report/case studies (n = 4)

(Crisan et al., 2022; Ferguson & Damluji,1988; Rania et al., 2021; Yum et al., 2009).

**References**

Appolinario, J. C., Sichieri, R., Lopes, C. S., Moraes, C. E., da Veiga, G. V., Freitas, S., Nunes, M. A. A., Wang, Y.-P., & Hay, P. (2022). Correlates and impact of DSM-5 binge eating disorder, bulimia nervosa and recurrent binge eating: A representative population survey in a middle-income country*. SOCIAL PSYCHIATRY AND PSYCHIATRIC EPIDEMIOLOGY, 57*(7), 1491–1503. https://doi.org/10.1007/s00127-022-02223-z

Aragona, M., Petta, A. M., & Balbi, A. (2015). Psychotic phenomena in Binge Eating Disorder: an exploratory MMPI-2 study. *Arch Psychiatry Psychotherapy, 17*(2), 13-20.

Ashour, A. A., Fahmi, M. K., Mohamed, R. N., Basha, S., Binmadi, N., Enan, E. T., Basalim, A., & Al Qahatani, A. (2022). Association between gastric reflux, obesity and erosive tooth wear among psychiatric patients. *MEDICINE*, *101*(7). https://doi.org/10.1097/MD.0000000000028923

Berthold, N., Pytte, J., Bulik, C. M., Tschochner, M., Medland, S. E., & Akkari, P. A. (2022). Bridging the gap: Short structural variants in the genetics of anorexia nervosa. *INTERNATIONAL JOURNAL OF EATING DISORDERS*, *55*(6), 747–753. <https://doi.org/10.1002/eat.23716>

Bou Khalil R. (2021). Targeting all psychopathological dimensions in the treatment of anorexia nervosa*. L'Encephale, 47*(1), 79–81. https://doi.org/10.1016/j.encep.2020.06.002

Brancati, G. E., Barbuti, M., Calderone, A., Fierabracci, P., Salvetti, G., Weiss, F., Santini, F., & Perugi, G. (2022). Prevalence and psychiatric comorbidities of night-eating behavior in obese bariatric patients: Preliminary evidence for a connection between night-eating and bipolar spectrum disorders. *EATING AND WEIGHT DISORDERS-STUDIES ON ANOREXIA BULIMIA AND OBESITY*, *27*(5), 1695–1704. <https://doi.org/10.1007/s40519-021-01306-1>

Bühren, K., Schwarte, R., Fluck, F., Timmesfeld, N., Krei, M., Egberts, K., Pfeiffer, E., Fleischhaker, C., Wewetzer, C., & Herpertz-Dahlmann, B. (2014). Comorbid psychiatric disorders in female adolescents with first-onset anorexia nervosa. *European eating disorders review : the journal of the Eating Disorders Association, 22*(1), 39–44. https://doi.org/10.1002/erv.2254

Bunting, L., McCartan, C., Davidson, G., Grant, A., Mulholland, C., Schubotz, D., McBride, O., Murphy, J., & Shevlin, M. (2022). Rationale and methods of the 'Northern Ireland Youth Wellbeing Survey' and initial findings from the Strengths and Difficulties Questionnaire. *Clinical child psychology and psychiatry, 27*(3), 670–685. https://doi.org/10.1177/13591045221075525

Cao, H., Wang, J., Baranova, A., & Zhang, F. (2022). Classifying major mental disorders genetically. *Progress in Neuro-Psychopharmacology & Biological Psychiatry*, *112*(q45, 8211617), 110410. <https://doi.org/10.1016/j.pnpbp.2021.110410>

Crișan, C., Androne, B., Barbulescu, L. D., & Suciu, B. D. (2022). Nexus of Delusions and Overvalued Ideas: A Case of Comorbid Schizophrenia and Anorexia in the View of the New ICD-11 Classification System. *The American journal of case reports, 23,* e933759. https://doi.org/10.12659/AJCR.933759

de Beaurepaire R. (2021). Binge Eating Disorders in Antipsychotic-Treated Patients With Schizophrenia: Prevalence, Antipsychotic Specificities, and Changes Over Time*. Journal of clinical psychopharmacology, 41*(2), 114–120. https://doi.org/10.1097/JCP.0000000000001357

de Jonge, P., Wardenaar, K. J., Lim, C. C., Aguilar-Gaxiola, S., Alonso, J., Andrade, L. H., ... & Scott, K. (2018). The cross-national structure of mental disorders: results from the World Mental Health Surveys*. Psychological medicine, 48*(12), 2073-2084.

De Young, K., & Rettler, L. (2023). Causal Connections Between Anorexia Nervosa and Delusional Beliefs. *Review of Philosophy and Psychology,* 1-22.

Diaz-Marsa, M., Pemau, A., de la Torre-luque, A., Vaz-Leal, F., Rojo-Moreno, L., Beato-Fernandez, L., Graell, M., Carrasco-Diaz, A., & Carrasco, J. (2023). Executive dysfunction in eating disorders: Relationship with clinical features. *PROGRESS IN NEURO-PSYCHOPHARMACOLOGY & BIOLOGICAL PSYCHIATRY, 120*. https://doi.org/10.1016/j.pnpbp.2022.110649

Ding, H., Ouyang, M., Wang, J., Xie, M., Huang, Y., Yuan, F., Jia, Y., Zhang, X., Liu, N., & Zhang, N. (2022). Shared genetics between classes of obesity and psychiatric disorders: A large-scale genome-wide cross-trait analysis*. JOURNAL OF PSYCHOSOMATIC RESEARCH*, 162. <https://doi.org/10.1016/j.jpsychores.2022.111032>

Eagles, J. M., Wilson, A. M., Hunter, D., & Callender, J. S. (1990). A comparison of anorexia nervosa and affective psychosis in young females. *Psychological medicine, 20*(1), 119–123. https://doi.org/10.1017/s0033291700013295

Ferguson, J. M., & Damluji, N. F. (1988). Anorexia nervosa and schizophrenia*. International Journal of Eating Disorders, 7*(3), 343-352.

Flynn, R., Massion, T., Kosmas, J., Smith, S., Mastronardi, C., & Graham, A. (2023). Positive affect dysregulation and its relation to binge eating size and frequency. *FRONTIERS IN PSYCHOLOGY*, *14*. <https://doi.org/10.3389/fpsyg.2023.1146549>

Frigaard, J., Hynne, H., Randsborg, K., Mellin-Olsen, T., Young, A., Rykke, M., Singh, P., Hove, L., Hofgaard, A., & Jensen, J. (2023). Exploring oral health indicators, oral health-related quality of life and nutritional aspects in 23 medicated patients from a short-term psychiatric ward. *FRONTIERS IN PUBLIC HEALTH, 11*. https://doi.org/10.3389/fpubh.2023.1083256

Ganson, K. T., Cuccolo, K., & Nagata, J. M. (2022). Associations between psychosis symptoms and eating disorders among a national sample of U.S. college students. *Eating Behaviors*, *45*(101090048), 101622. <https://doi.org/10.1016/j.eatbeh.2022.101622>

Horsager, C., Faerk, E., Gearhardt, A. N., Lauritsen, M. B., & Ostergaard, S. D. (2022). Food addiction comorbid to mental disorders in adolescents: A nationwide survey and register-based study. *Eating and Weight Disorders : EWD, 27*(3), 945–959. https://doi.org/10.1007/s40519-021-01212-6

Hsu, L. K., Meltzer, E. S., & Crisp, A. H. (1981). Schizophrenia and anorexia nervosa. *The Journal of nervous and mental disease, 169*(5), 273–276. https://doi.org/10.1097/00005053-198105000-00001

Hubel, C., Abdulkadir, M., Herle, M., Loos, R. J. F., Breen, G., Bulik, C. M., & Micali, N. (2021). One size does not fit all. Genomics differentiates among anorexia nervosa, bulimia nervosa, and binge-eating disorder. *The International Journal of Eating Disorders*, *54*(5), 785–793. <https://doi.org/10.1002/eat.23481>

John, A., Friedmann, Y., DelPozo-Banos, M., Frizzati, A., Ford, T., & Thapar, A. (2022). Association of school absence and exclusion with recorded neurodevelopmental disorders, mental disorders, or self-harm: a nationwide, retrospective, electronic cohort study of children and young people in Wales, UK*. The lancet. Psychiatry, 9*(1), 23–34. <https://doi.org/10.1016/S2215-0366(21)00367-9>

Johansson, T., Birgegard, A., Zhang, R., Bergen, S. E., Landen, M., Petersen, L., V., Bulik, C. M., & Hubel, C. (2022). Polygenic association with severity and long-term outcome in eating disorder cases*. TRANSLATIONAL PSYCHIATRY, 12*(1). https://doi.org/10.1038/s41398-022-01831-2

Kambanis, P. E., Harshman, S. G., Kuhnle, M. C., Kahn, D. L., Dreier, M. J., Hauser, K., Slattery, M., Becker, K. R., Breithaupt, L., Misra, M., Micali, N., Lawson, E. A., Eddy, K. T., & Thomas, J. J. (2022). Differential comorbidity profiles in avoidant/restrictive food intake disorder and anorexia nervosa: Does age play a role? *International Journal of Eating Disorders*, *55*(10), 1397–1403. <https://doi.org/10.1002/eat.23777>

Kendler, K. S., Ohlsson, H., Sundquist, J., & Sundquist, K. (2021). The patterns of family genetic risk scores for eleven major psychiatric and substance use disorders in a Swedish national sample*. Translational Psychiatry, 11*(1), 326. <https://doi.org/10.1038/s41398-021-01454-z>

Khazaal, Y., Billieux, J., Fresard, E., Huguelet, P., Van der Linden, M., & Zullino, D. (2010). A measure of dysfunctional eating-related cognitions in people with psychotic disorders. *The Psychiatric quarterly, 81*(1), 49–56. https://doi.org/10.1007/s11126-009-9117-3

Kim, M., Yang, S., Kim, H., Jo, A., Jhon, M., Lee, J., Ryu, S., Kim, J., Kweon, Y., & Kim, S. (2023). Effects of Dietary Habits on General and Abdominal Obesity in Community-dwelling Patients with Schizophrenia. *CLINICAL PSYCHOPHARMACOLOGY AND NEUROSCIENCE, 21*(2), 68–76. https://doi.org/10.9758/cpn.2023.21.1.68

Kouidrat, Y., Amad, A., Stubbs, B., Louhou, R., Renard, N., Diouf, M., Lalau, J. D., & Loas, G. (2018). Disordered eating behaviors as a potential obesogenic factor in schizophrenia. *Psychiatry research, 269,* 450–454. https://doi.org/10.1016/j.psychres.2018.08.083

Kurpad, S. S., George, S. A., & Srinivasan, K. (2010). Binge eating and other eating behaviors among patients on treatment for psychoses in India*. Eating and weight disorders : EWD, 15*(3), e136–e143. https://doi.org/10.1007/BF03325293

Lasson, C., & Raynal, P. (2021). Personality profiles in young adults with orthorexic eating behaviors. *EATING AND WEIGHT DISORDERS-STUDIES ON ANOREXIA BULIMIA AND OBESITY*, *26*(8), 2727–2736. <https://doi.org/10.1007/s40519-021-01124-5>

Lee, Y., Lee, D., Jung, H., Cho, Y., Baek, J., & Hong, K. (2022). Heterogeneous early illness courses of Korean patients with bipolar disorders: Replication of the staging model. *BMC PSYCHIATRY, 22*(1). https://doi.org/10.1186/s12888-022-04318-y

Lee, Y. Y., Lau, J. H., Seet, V., Whitton, C., Asharani, P., Kumar, F. D. S., Wang, P., Roystonn, K., Cetty, L., Teh, W. L., Verma, S., Mok, Y. M., & Subramaniam, M. (2021). Dietary intake of persons with depressive and psychotic disorders in Singapore. *ANNALS ACADEMY OF MEDICINE SINGAPORE, 50*(5), 379–389. https://doi.org/10.47102/annals-acadmedsg.2020585

Legg, N., Robillard, C., & Turner, B. (2023). Examining the components and stability of negative affect in disordered eating frequency. *EUROPEAN EATING DISORDERS REVIEW*. <https://doi.org/10.1002/erv.2990>

Leutner, M., Dervic, E., Bellach, L., Klimek, P., Thurner, S., & Kautzky, A. (2023). Obesity as pleiotropic risk state for metabolic and mental health throughout life*. TRANSLATIONAL PSYCHIATRY, 13*(1). https://doi.org/10.1038/s41398-023-02447-w

Ling, N. C. Y., Serpell, L., Burnett-Stuart, S., & Pugh, M. (2022). Interviewing anorexia: How do individuals given a diagnosis of anorexia nervosa experience Voice Dialogue with their eating disorder voice? A qualitative analysis. *Clinical Psychology & Psychotherapy*, *29*(2), 600–610. https://doi.org/10.1002/cpp.2652

Liu, C., Liu, S., Wang, X., & Ji, Y. (2021). Neuropsychiatric profiles in mild cognitive impairment with Lewy bodies. *Aging & Mental Health*, *25*(11), 2011–2017. https://doi.org/10.1080/13607863.2020.1817311

Lu, C., Jin, D., Palmer, N., Fox, K., Kohane, I. S., Smoller, J. W., & Yu, K.-H. (2022). Large-scale real-world data analysis identifies comorbidity patterns in schizophrenia. *Translational Psychiatry*, *12*(1), 154. <https://doi.org/10.1038/s41398-022-01916-y>

Lynham, A. J., Jones, I. R., & Walters, J. T. R. (2022). Web-Based Cognitive Testing in Psychiatric Research: Validation and Usability Study. *JOURNAL OF MEDICAL INTERNET RESEARCH, 24*(2). https://doi.org/10.2196/28233

McElroy, S. L., Crow, S., Biernacka, J. M., Winham, S., Geske, J., Barboza, A. B. C., ... & Frye, M. A. (2013). Clinical phenotype of bipolar disorder with comorbid binge eating disorder. *Journal of affective disorders, 150*(3), 981-986.

Mereu, A., Fantoni, T., Caini, S., Monzali, F., Roselli, E., Taddei, S., Lucarelli, S., & Pisano, T. (2022). Suicidality in adolescents with onset of anorexia nervosa. *Eating and Weight Disorders : EWD*, *27*(7), 2447–2457. https://doi.org/10.1007/s40519-022-01384-9

Motteli, S., Provaznikova, B., Vetter, S., Jager, M., Seifritz, E., & Hotzy, F. (2023). Examining Nutrition Knowledge, Skills, and Eating Behaviours in People with Severe Mental Illness: A Cross-Sectional Comparison among Psychiatric Inpatients, Outpatients, and Healthy Adults. *NUTRIENTS*, *15*(9). https://doi.org/10.3390/nu15092136

Muzi, L., Tieghi, L., Rugo, M. A., & Lingiardi, V. (2021). Personality as a predictor of symptomatic change in a residential treatment setting for anorexia nervosa and bulimia nervosa. *Eating and Weight Disorders : EWD*, *26*(4), 1195–1209. <https://doi.org/10.1007/s40519-020-01023-1>

Neumann, A., Nolte, I. M., Pappa, I., Ahluwalia, T. S., Pettersson, E., Rodriguez, A., Whitehouse, A., van Beijsterveldt, C. E. M., Benyamin, B., Hammerschlag, A. R., Helmer, Q., Karhunen, V., Krapohl, E., Lu, Y., van der Most, P. J., Palviainen, T., St Pourcain, B., Seppala, I., Suarez, A., … Tiemeier, H. (2022). A genome-wide association study of total child psychiatric problems scores. *PloS One, 17*(8), e0273116. <https://doi.org/10.1371/journal.pone.0273116>

Niwinski, P., Remberk, B., Rybakowski, F., & Rokicki, D. (2021). Psychiatric Symptoms as the First or Solitary Manifestation of Somatic Illnesses: Hyperammonaemia Type II. *NEUROPSYCHOBIOLOGY, 80*(3), 271–275. https://doi.org/10.1159/000508679

Olgiati, P., Fanelli, G., Atti, A., De Ronchi, D., & Serretti, A. (2022). Clinical correlates and prognostic impact of binge-eating symptoms in major depressive disorder. *INTERNATIONAL CLINICAL PSYCHOPHARMACOLOGY, 37*(6), 247–254. <https://doi.org/10.1097/YIC.0000000000000422>

Oswalt, S. B., Lederer, A. M., Chestnut-Steich, K., Day, C., Halbritter, A., & Ortiz, D. (2020). Trends in college students' mental health diagnoses and utilization of services, 2009-2015*. Journal of American college health : J of ACH, 68*(1), 41–51. https://doi.org/10.1080/07448481.2018.1515748

Pedram, P., Patten, S. B., Bulloch, A. G. M., Williams, J. V. A., & Dimitropoulos, G. (2021). Self-Reported Lifetime History of Eating Disorders and Mortality in the General Population: A Canadian Population Survey with Record Linkage. *NUTRIENTS, 13*(10). <https://doi.org/10.3390/nu13103333>

Phillipou, A., Tan, E. J., Toh, W. L., Van Rheenen, T. E., Meyer, D., Neill, E., Sumner, P., & Rossell, S. L. (2021). Mental health of individuals with and without eating disorders across six months and two waves of COVID-19. *EATING BEHAVIORS, 43*. https://doi.org/10.1016/j.eatbeh.2021.101564

Polushina, T., Banerjee, N., Giddaluru, S., Bettella, F., Espeseth, T., Lundervold, A. J., Djurovic, S., Cichon, S., Hoffmann, P., Nöthen, M. M., Steen, V. M., Andreassen, O. A., & Le Hellard, S. (2021). Identification of pleiotropy at the gene level between psychiatric disorders and related traits*. Translational psychiatry, 11*(1), 410. <https://doi.org/10.1038/s41398-021-01530-4>

Rania, M., de Filippis, R., Caroleo, M., Carbone, E., Aloi, M., Bratman, S., & Segura-Garcia, C. (2021). Pathways to orthorexia nervosa: a case series discussion. *Eating and weight disorders : EWD, 26*(5), 1675–1683. https://doi.org/10.1007/s40519-020-00948-x

Rasmussen, A. R., Reich, D., Lavoie, S., Li, E., Hartmann, J. A., McHugh, M., ... & Nelson, B. (2020). The relation of basic self‐disturbance to self‐harm, eating disorder symptomatology and other clinical features: Exploration in an early psychosis sample. *Early intervention in psychiatry, 14*(3), 275-282.

Regeneron Genetics Ctr, Kember, R. L., Merikangas, A. K., Verma, S. S., Verma, A., Judy, R., Damrauer, S. M., Ritchie, M. D., Rader, D. J., & Bucan, M. (2021). Polygenic Risk of Psychiatric Disorders Exhibits Cross-trait Associations in Electronic Health Record Data From European Ancestry Individuals. *BIOLOGICAL PSYCHIATRY*, *89*(3), 236–245. <https://doi.org/10.1016/j.biopsych.2020.06.026>

Remberk, B., Niwinski, P., Brzoska-Konkol, E., Borowska, A., Papasz-Siemieniuk, A., Bragoszewska, J., Bazynska, A. K., Szostakiewicz, L., & Herman, A. (2021). Ectodermal disturbance in development shared by anorexia and schizophrenia may reflect neurodevelopmental abnormalities. *Brain and Behavior, 11*(10), e2281. https://doi.org/10.1002/brb3.2281

Risch, L., Hotzy, F., Vetter, S., Hiller, S., Wallimann, K., Seifritz, E., & Motteli, S. (2023). Assessment of Nutritional Status and Risk of Malnutrition Using Adapted Standard Tools in Patients with Mental Illness and in Need of Intensive Psychiatric Treatment. *INTERNATIONAL JOURNAL OF ENVIRONMENTAL RESEARCH AND PUBLIC HEALTH*, *20*(1). <https://doi.org/10.3390/ijerph20010109>

Sahlan, R., & Sala, M. (2022). Eating disorder psychopathology and negative affect in Iranian college students: A network analysis. *JOURNAL OF EATING DISORDERS*, *10*(1). <https://doi.org/10.1186/s40337-022-00683-x>

Sankaranarayanan, A., Johnson, K., Mammen, S. J., Wilding, H. E., Vasani, D., Murali, V., Mitchison, D., Castle, D. J., & Hay, P. (2021). Disordered Eating among People with Schizophrenia Spectrum Disorders: A Systematic Review. *Nutrients, 13*(11), Article 11. https://doi.org/10.3390/nu13113820

Sellbom, M., Forbush, K. T., Gould, S. R., Markon, K. E., Watson, D., & Witthoft, M. (2022). HiTOP Assessment of the Somatoform Spectrum and Eating Disorders. *ASSESSMENT*, *29*(1), 62–74. https://doi.org/10.1177/10731911211020825

Sevillano-Jimenez, A., Romero-Saldana, M., Garcia-Rodriguez, M., Molina-Luque, R., & Molina-Recio, G. (2022). Nutritional Impact and Eating Pattern Changes in Schizophrenic Spectrum Disorders after Health Education Program on Symbiotic Dietary Modulation Offered by Specialised Psychiatric Nursing-Two-Arm Randomised Clinical Trial. *NUTRIENTS*, *14*(24). <https://doi.org/10.3390/nu14245388>

Small, A. C., Madero, J., Gross, H., Teagno, L., Leib, J., & Ebert, M. (1981). A comparative analysis of primary anorexics and schizophrenics on the MMPI. *Journal of Clinical Psychology, 37*(4), 733-736.

Solmi, F., Mascarell, M. C., Zammit, S., Kirkbride, J. B., & Lewis, G. (2019). Polygenic risk for schizophrenia, disordered eating behaviours and body mass index in adolescents*. The British journal of psychiatry : the journal of mental science, 215(*1), 428–433. https://doi.org/10.1192/bjp.2019.39

Straface, E., De Jacobis, I., Capriati, T., Pretelli, I., Grandin, A., Mascolo, C., Vona, R., Gambardella, L., Cittadini, C., Villani, A., & Marchili, M. (2023). The impact of the COVID-19 pandemic on eating disorders risk and symptoms: A retrospective study. *ITALIAN JOURNAL OF PEDIATRICS*, *49*(1). <https://doi.org/10.1186/s13052-023-01443-6>

Striegel‐Moore, R. H., Garvin, V., Dohm, F. A., & Rosenheck, R. A. (1999). Eating disorders in a national sample of hospitalized female and male veterans: Detection rates and psychiatric comorbidity. *International Journal of Eating Disorders*, *25*(4), 405-414. <https://onlinelibrary.wiley.com/doi/10.1002/(SICI)1098-108X(199905)25:4%3C405::AID-EAT5%3E3.0.CO;2-F>

Yazdi, A. S. H., Eslamzadeh, M., Mohammadi, M. R., Khaleghi, A., Hooshyari, Z., Moharreri, F., ... & Ashouri, S. (2020). A survey of psychiatric disorders and their comorbidities in children and adolescents*. Galen Medical Journal, 9,* e1714.

Yum, S. Y., Caracci, G., & Hwang, M. Y. (2009). Schizophrenia and eating disorders. *The Psychiatric clinics of North America, 32*(4), 809–819. <https://doi.org/10.1016/j.psc.2009.09.004>

Wainberg, Mkendler., Jacobs, G. R., Voineskos, A. N., & Tripathy, S. J. (2022). Neurobiological, familial and genetic risk factors for dimensional psychopathology in the Adolescent Brain Cognitive Development study. *Molecular Psychiatry, 27*(6), 2731–2741. https://doi.org/10.1038/s41380-022-01522-w

Wattick, R., Olfert, M., Hagedorn-Hatfield, R., Barr, M., Claydon, E., & Brode, C. (2023). Diet quality and eating behaviors of college-attending young adults with food addiction. *EATING BEHAVIORS, 49*. <https://doi.org/10.1016/j.eatbeh.2023.101710>

Wojtaszek, J. A., & Saules, K. K. (n.d.). The moderating effects of intolerance of uncertainty and social connectedness on college students’ addictive behaviors and mental health symptoms during the COVID-19 pandemic*. JOURNAL OF AMERICAN COLLEGE HEALTH.* https://doi.org/10.1080/07448481.2022.2082848

**Appendix E: Risk of biases and quality assessment**

The risk of bias and the methodological quality of the selected studies according to Newcastle-Ottawa Quality Scale (NOS), are summarised in the below tables.

**Table 8**

*Quality assessment of cross-sectional studies*

| **First author,**  **year** | | **Sample representative-ness (eligibility, sample size, random sampling)**  **(0-2)** | **Exposure assessment (is the topic well-described?) (0-1)** | **Comparability of groups (match and adjustment for confounding factors)**  **(0-2)** | **Outcome assessment (tools, blinding, correct recording)**  **(0-2)** | **Outcome appropria-tely reported/**  **statistical tests (0-1)** | **Total score (0-8)** |
| --- | --- | --- | --- | --- | --- | --- | --- |
| Blinder, 2006 | 2 | | 1 | 2 | 2 | 1 | 8 |
| Catone, 2021 | 2 | | 1 | 2 | 1 | 1 | 7 |
| Convertino, 2022 | 1 | | 1 | 2 | 2 | 1 | 7 |
| Civil Arslan, 2015 | 2 | | 1 | 1 | 2 | 1 | 7 |
| Herzog, 1992 | 2 | | 1 | 1 | 2 | 1 | 7 |
| Hudson, 1984 | 2 | | 1 | 0 | 1 | 1 | 5 |
| Kambanis, 2023 | 1 | | 1 | 2 | 2 | 1 | 7 |
| Konstantakopoulos, 2012 | 2 | | 1 | 2 | 2 | 1 | 8 |
| Koyanagi, 2016 | 2 | | 1 | 2 | 1 | 1 | 7 |
| Malaspina, 2019 | 2 | | 1 | 2 | 2 | 1 | 8 |
| Malcolm, 2022 | 1 | | 1 | 2 | 1 | 1 | 6 |
| McGrath, 2016 | 1 | | 1 | 2 | 2 | 1 | 7 |
| Mensi, 2020 | 2 | | 1 | 1 | 2 | 1 | 7 |
| Mohammadi, 2020 | 2 | | 1 | 1 | 1 | 1 | 6 |
| Mutiso, 2022 | 2 | | 1 | 2 | 1 | 1 | 7 |
| Patel, 2018 | 2 | | 1 | 1 | 2 | 1 | 7 |
| Rodgers, 2022 | 2 | | 1 | 2 | 1 | 1 | 7 |
| Palmese, 2013 | 1 | | 1 | 2 | 2 | 1 | 7 |
| Salazar de Pablo, 2020 | 2 | | 1 | 2 | 2 | 1 | 8 |
| Saraçli, 2015 | 2 | | 1 | 1 | 2 | 1 | 7 |
| Stein, 2005 | 2 | | 1 | 1 | 2 | 1 | 7 |
| Steinglass, 2007 | 1 | | 1 | 1 | 2 | 1 | 6 |
| Teh, 2021 | 2 | | 1 | 2 | 2 | 1 | 8 |
| Valente, 2017 | 2 | | 1 | 0 | 2 | 1 | 6 |

**Table 9**

*Quality assessment of case control studies*

| **First author,**  **year** | **Case selection and definition (representativeness) (0-2)** | **Control selection and definition (0-2)** | **Group comparability (match and adjustment)**  **(0-2)** | **Exposure assessment method (tools, explanation, is it reported properly?)**  **(0-2)** | **Total score**  **(0-8)** |
| --- | --- | --- | --- | --- | --- |
| Camprodon-Boadas, 2023 | 1 | 1 | 1 | 2 | 5 |
| Fawzi, 2012 | 2 | 2 | 2 | 2 | 8 |
| Grilo, 1996 | 2 | 2 | 1 | 1 | 6 |
| Khazaal, 2006 | 2 | 2 | 1 | 2 | 7 |
| Khosravi, 2020 | 1 | 2 | 2 | 2 | 7 |
| Konstantakopoulos, 2020 | 2 | 1 | 2 | 2 | 7 |
| Lyketsos, 1985 | 2 | 1 | 0 | 2 | 5 |
| Lysaker, 2023 | 2 | 2 | 2 | 2 | 8 |
| Miotto, 2010 | 2 | 2 | 2 | 2 | 8 |
| Mountjoy, 2014 | 1 | 1 | 1 | 2 | 5 |
| Striegel-Moore, 1999 | 2 | 2 | 2 | 1 | 7 |

**Table 10**

*Quality assessment of cohort studies*

| **First author, Year** | **Sample representativeness (eg total population, random group, selected group)**  **(0-1)** | **Exposure method (is it robust to assess exposure?) (0-1)** | **Group comparability (match and adjustment for confounding factors)**  **(0-2)** | **Assessment of outcome (tools, blinding) (0-2)** | **Follow-up period (is it sufficient for outcomes to occur?)**  **(0-1)** | **Loss to follow-up (<30%) (0-1)** | **Total score**  **(0-8)** |
| --- | --- | --- | --- | --- | --- | --- | --- |
| De Young, 2022 | 0 | 1 | 2 | 2 | 1 | 1 | 7 |
| Fekih-Romdhane, 2024 | 0 | 1 | 2 | 1 | 1 | 0 | 5 |
| Plana-Ripoll, 2019 | 1 | 1 | 2 | 1 | 1 | 1 | 7 |
| Pruccoli, 2023 | 1 | 1 | 1 | 2 | 1 | 1 | 7 |
| Rarmussen, 2019 | 0 | 1 | 1 | 2 | 1 | 1 | 6 |
| Salvatore, 2021 | 1 | 1 | 1 | 2 | 1 | 1 | 7 |
| Solmi, 2018 | 1 | 1 | 2 | 2 | 1 | 0 | 7 |
| Zhang, 2020 | 1 | 1 | 2 | 2 | 1 | 1 | 8 |
| Zhang, 2023 | 1 | 1 | 1 | 2 | 1 | 1 | 7 |

# **Appendix F: *Forest plot showing comorbidity of eating disorders and psychotic disorders in clinical samples with eating disorders***


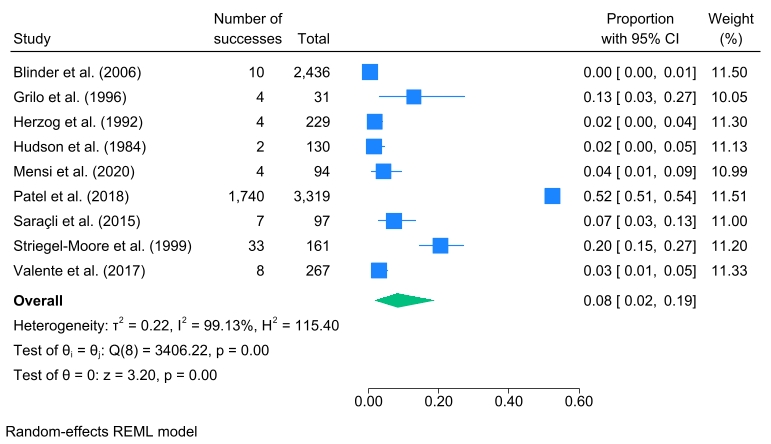


# **Appendix G: *Forest plot showing comorbidity of eating disorders and psychotic disorders in clinical samples with psychosis***


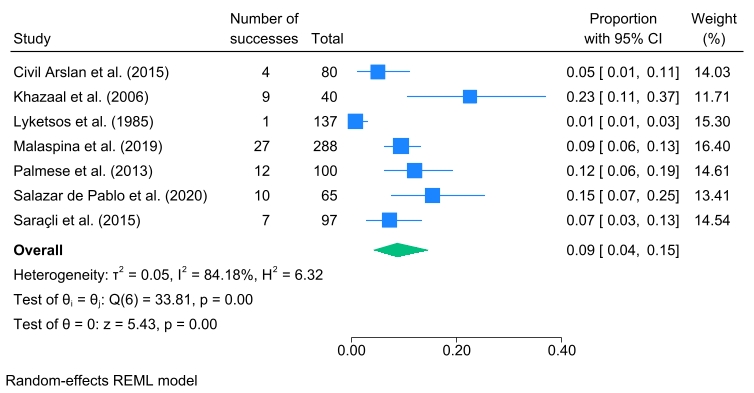


# **Appendix H: Forest plot for meta-analysis of the comorbidity between eating disorders and psychotic disorders across North American studies**


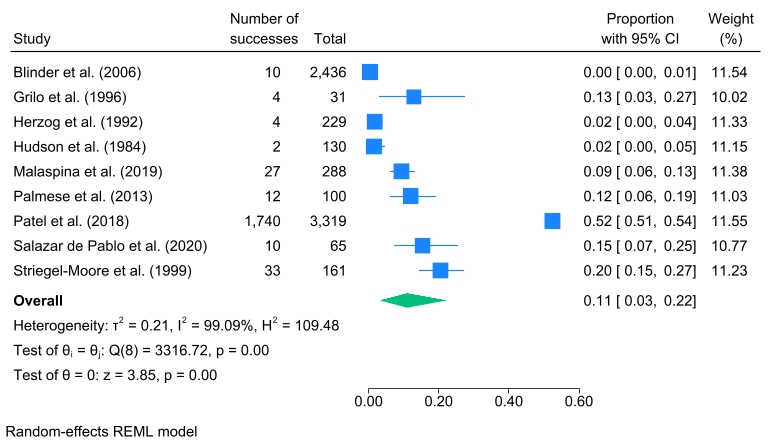


# **Appendix I: Forest plot for meta-analysis of the comorbidity between eating disorders and psychotic disorders across European studies**


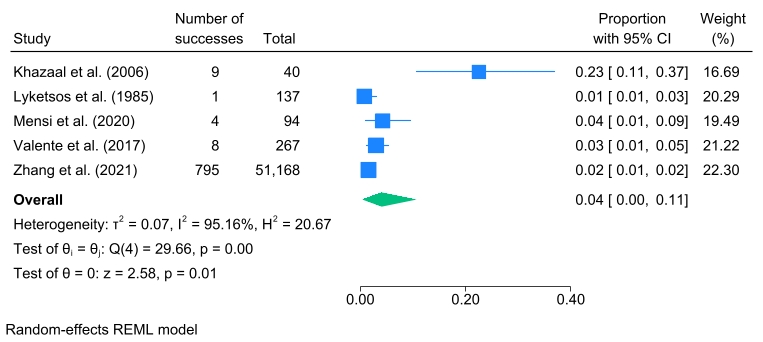


# **Appendix J: Forest plot for meta-analysis of the comorbidity between eating disorders and psychotic disorders across cross-sectional studies**


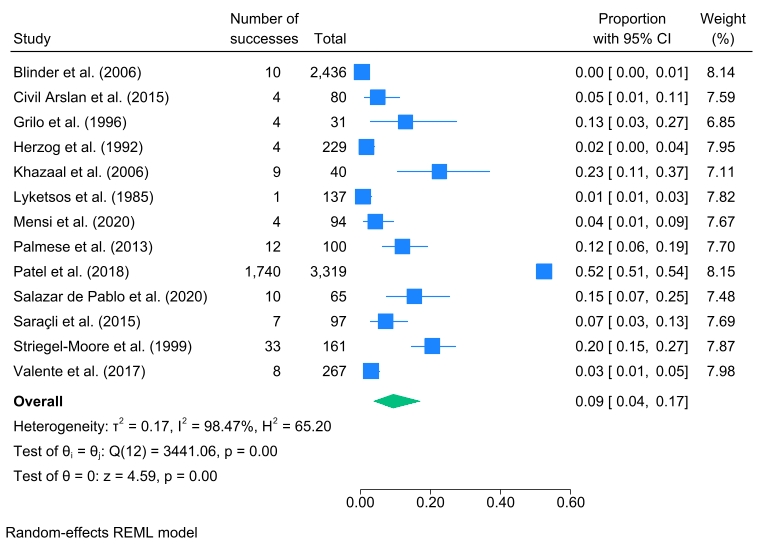


# **Appendix K: Forest plot for meta-analysis of the comorbidity between eating disorders and psychotic disorders across retrospective studies**


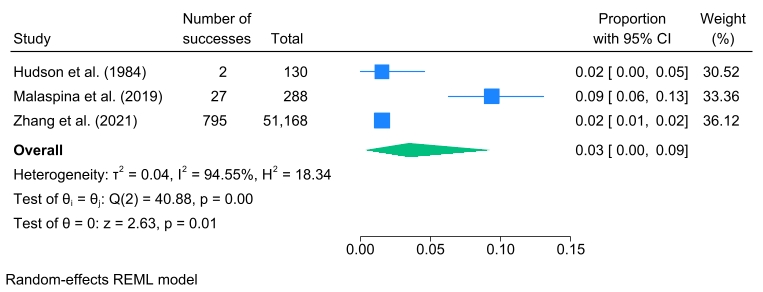


# **Appendix L: Funnel plot for meta-analysis of the comorbidity between eating disorders and psychotic disorders across clinical and general population samples (*k* = 16; *p* value for Egger’s test = 0.58)**


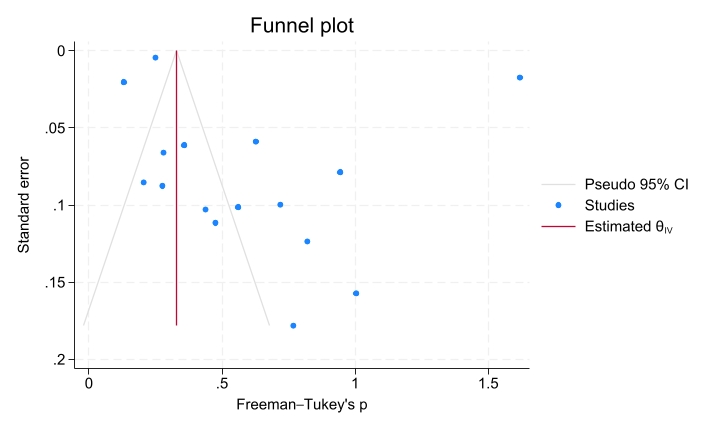


# **Appendix M: Funnel plot for meta-analysis of the comorbidity between eating disorders and psychotic disorders across clinical populations with eating disorders (*k* = 9; *p* value for Egger’s test = 0.76)**

**
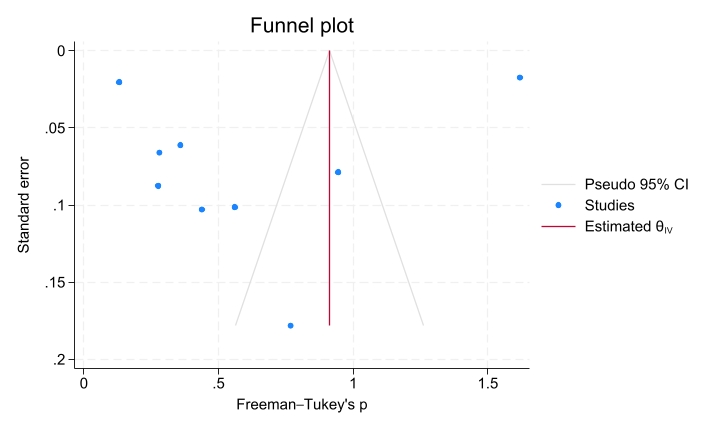
**

# **Appendix N: Funnel plot for meta-analysis of the comorbidity between eating disorders and psychotic disorders across clinical populations with psychosis (k = 7; *p* value for Egger’s test = 0.12)**


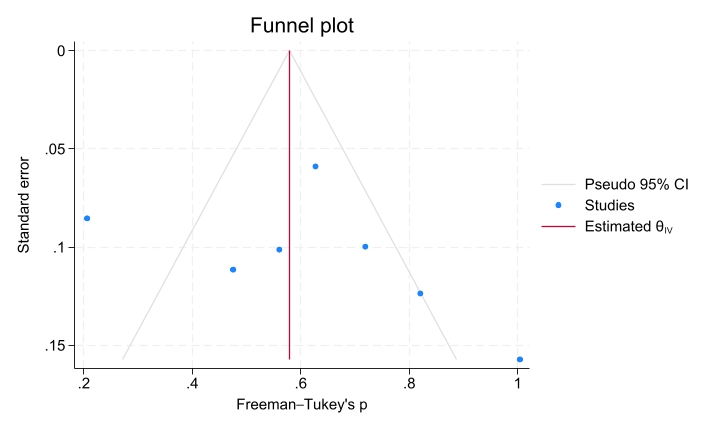


# **Appendix O: Funnel plot for meta-analysis of the comorbidity between eating disorders and psychotic disorders across North American studies (*k* = 9; *p* value for Egger’s test = 0.90)**


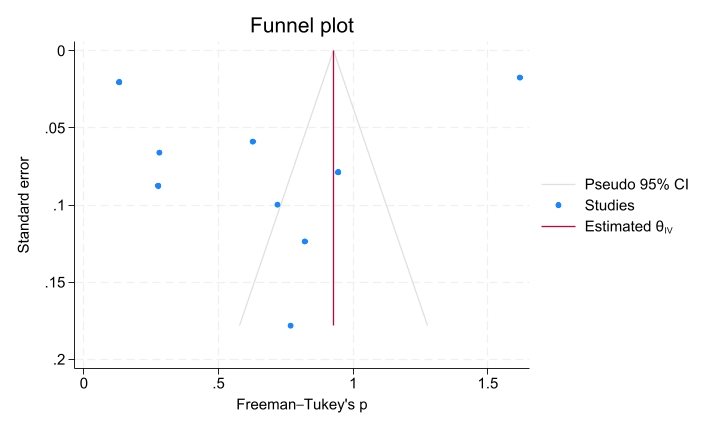


# **Appendix P: Funnel plot for meta-analysis of the comorbidity between eating disorders and psychotic disorders across European studies (*k* = 5; *p* value for Egger’s test = 0.03)**


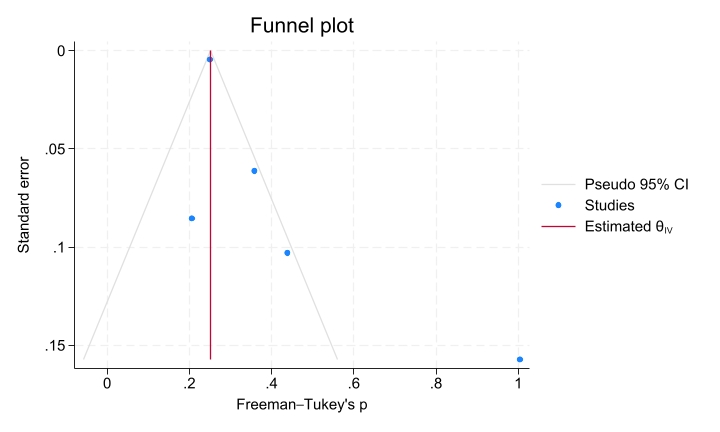


# **Appendix Q: Funnel plot for meta-analysis of the comorbidity between eating disorders and psychotic disorders across cross-sectional studies (*k* = 13; *p* value for Egger’s test = 0.88)**


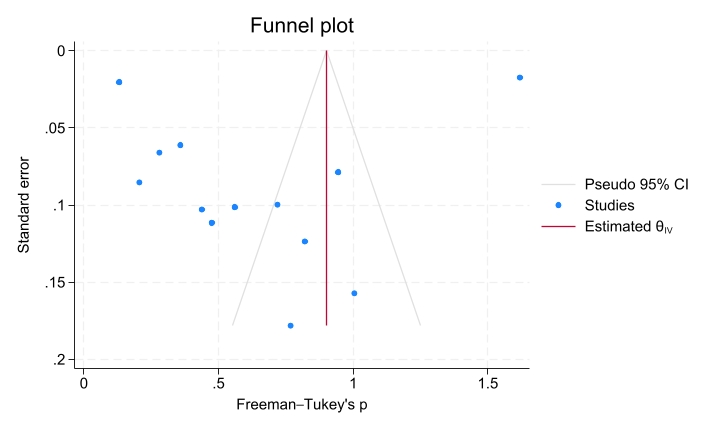


# **Appendix R: Funnel plot for meta-analysis of the comorbidity between eating disorders and psychotic disorders across retrospective studies (k = 3; *p* value for Egger’s test = 0.79)**


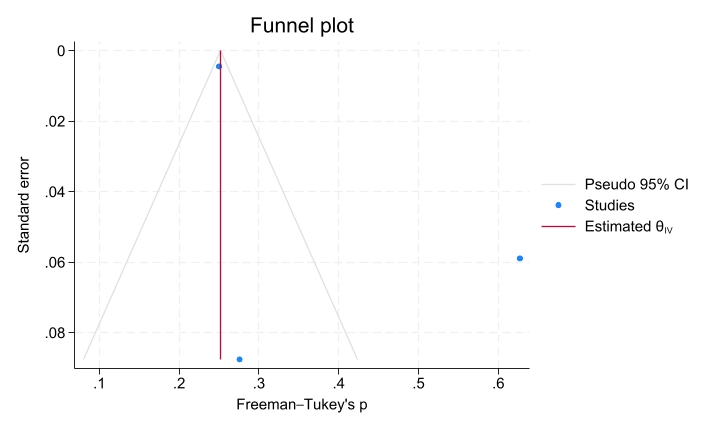

Supplement: Drymonitou et al. supplementary material [file S003329172500114Xsup001.docx]
